# Can a simulation-based training program impact the use of evidence based routine practices at birth? Results of a hospital-based cluster randomized trial in Mexico

**DOI:** 10.1371/journal.pone.0172623

**Published:** 2017-03-20

**Authors:** Jimena Fritz, Dilys M. Walker, Susanna Cohen, Gustavo Angeles, Hector Lamadrid-Figueroa

**Affiliations:** 1 Division of Reproductive Health, Research Center for Population Health, National Institute of Public Health (INSP), Cuernavaca, Morelos, México; 2 Department of Obstetrics, Gynecology and Reproductive Sciences, Bixby Center for Global Reproductive Health, University of California in San Francisco, (UCSF), San Francisco, California, United States of America; 3 College of Nursing, University of Utah, Salt Lake City, Utah, United States of America; 4 Department of Maternal and Child Health, University of North Carolina at Chapel Hill (UNC), Chapel Hill, North Carolina, United States of America; TNO, NETHERLANDS

## Abstract

**Background:**

In Mexico, although the majority of births are attended in hospitals, reports have emerged of obstetric violence, use of unsafe practices, and failure to employ evidence-based practices (EBP). Recent attention has refocused global efforts towards provision of quality care that is both patient-centered and evidence-based. Scaling up of local interventions should rely on strong evidence of effectiveness.

**Objective:**

To perform a secondary analysis to evaluate the impact of a simulation and team-training program (PRONTO) on the performance of EBP in normal births.

**Methods:**

A pair-matched cluster randomized controlled trial of the intervention was designed to measure the impact of the program (PRONTO intervention) on a sample of 24 hospitals (12 hospitals received the PRONTO training and 12 served as controls) in the states of Chiapas, Guerrero, and Mexico. We estimated the impact of receiving the intervention on the probability of birth practices performance in a sample of 641 observed births of which 318 occurred in the treated hospitals and 323 occurred in control hospitals. Data was collected at 4 time points (baseline, 4^th^, 8^th^ and 12^th^ months after the training). Women were blinded to treatment allocation but observers and providers were not. Estimates were obtained by fitting difference-in-differences logistic regression models considering confounding variables. The trial is registered at clinicaltrials.gov: # NCT01477554.

**Results:**

Significant changes were found following the intervention. At 4 months post-intervention an increase of 20 percentage points (p.p.) for complete Active Management of Third Stage of Labor (AMTSL) (p = 0.044), and 16 p.p. increase for Skin-to-Skin Contact (p = 0.067); at 12 months a 25 p.p. increase of the 1st step of AMTSL (p = 0.026) and a 42 p.p. increase of Delayed Cord Clamping (p = 0.004); at 4 months a 30 (p = 0.001) and at 8 months a 22 (p = 0.010) p.p. decrease for Uterine Sweeping.

**Conclusions:**

The intervention has an impact on adopting EBP at birth, contributing to an increased quality of care. Long lasting impacts on these practices are possible if there were to be a widespread adoption of the training techniques including simulation, team-training and facilitated discussions regarding routine care.

## Introduction

Reducing maternal and neonatal mortality (MM and NM) have been global priorities for over two decades.[1,2] Considerable effort has focused on enhancing infrastructure, training of birth attendants, and improving emergency obstetric care in limited-resource settings.[1,2] In Mexico, the 2013 MM ratio Global Burden of Disease estimate was 54 deaths per 100,000 live births [3] with the majority of deaths due to obstetric emergencies, namely postpartum hemorrhage, preeclampsia and sepsis.[4] Mexico, has focused on increasing the access to facility-based birth attended by skilled professionals.[5] Although, 99.6% of births in Mexico were attended by skilled providers in hospitals,[6] 80% of maternal deaths took place inside a medical facility and 87% of the women who died received facility-based attention before their death.[4] For NM, increased efforts have resulted in a decreased rate of 7.2 per 1,000 live births, a figure that is still high compared to high-income countries.[7]

Global efforts have refocused towards the provision of patient-centered and evidence-based quality care, thus acknowledging that a good share of morbidity and mortality are linked to the mis-management of normal physiologic births and not only to the response to emergencies.[1,2,5,8] Renfrew and colleagues conducted a systematic review to categorize obstetric and neonatal practices by the level of evidence regarding benefit and/or harm.[1] They devised a framework for quality maternal and newborn care using the best available evidence for effective practices: continuous labour support, Active Management of Third Stage of Labor (AMTSL), Delayed umbilical Cord Clamping (DCC), Skin-to-Skin mother-baby Contact (SSC), restricted episiotomy, alternative vs. conventional institutional settings for birth, and others.[1] They identified several outcomes that could be improved by care within the scope of midwifery such as decreased number of unnecessary interventions and improved effective practices.[1,8,9]

Worldwide consensus establishes that three practices can improve outcomes and prevent significant childbirth complications, according to the World Health Organization (WHO) [10] and Mexican guidelines [11]: AMTSL, DCC, and immediate maternal-newborn SSC.

There is considerable evidence that AMTSL, prevents obstetric hemorrhage, justifying its practice with every birth.[8,9,10] DCC, has been shown to prevent anemia in the neonate for at least the first six months after birth.[12] SSC is linked to immediate breastfeeding and has numerous long-term physiologic benefits for both mother and baby, besides a lowered risk of postpartum hemorrhage.[13]

Despite global acceptance of these practices and their inclusion in national regulations, their routine implementation is rare in Mexico.[14,15,16] On the contrary, there are other potentially harmful practices whose routine use is pervasive in the Mexican health system, namely, routine episiotomy, fundal pressure (Kristeller maneuver) and uterine sweeping.

An episiotomy is a surgical incision through the perineum, performed for expediting the birth of the baby and was thought to prevent higher-level perineal lacerations.[17] The WHO, rejects the routine use of episiotomy and only recommends it for specific complications such as fetal distress, shoulder dystocia, or breech- presentation.[17,18] There is no evidence that routine episiotomy decreases perineal damage, future vaginal prolapse or urinary incontinence.[18] In fact, it is associated with an increase of third and fourth degree tears and subsequent anal sphincter muscle dysfunction, hematomas, infections, and pain.[18] Unfortunately, it continues to be a routine practice in Mexico, with some studies showing its use in 38% to 66% of all births.[14–16]

The Kristeller maneuver involves the application of manual pressure on the fundus of the uterus in an effort to expedite a vaginal birth.[19] It was historically used to avoid a prolonged second stage of labor or the need for cesarean section.[19] The maneuver itself carries increased risk of uterine rupture and other maternal morbidity, and as such it is not recommended as the risks outweigh the benefits.[19,20] Although few studies have tried to estimate its prevalence, the available evidence suggests it continues to be used routinely in Mexican institutions, with alarming figures ranging between 17% and 40%.[14]

In uterine sweeping, the provider inserts a gloved hand wrapped in gauze into the uterus after the birth of the placenta to remove any remaining placental parts.[21] The vast majority of placentas are expelled intact, thus negating the need for this as a routine prophylactic measure.[14,20] Additionally, this practice carries with it a number of risks including severe pain, infection, and uterine rupture.[14,20] The Kristeller maneuver continues to be routinely used throughout Mexico, as more than 80% of the women who received care in the states of Oaxaca,[14] and Guerrero,[16] received this intervention.

The current situation regarding these clinical practices points to some clear disconnects in the provision of care during physiologic births that may be important for the reduction of MM and NM.[9] Global programs for in-service and interprofessional training of providers can help to meet the need for continued education and practice of care during childbirth.[9] All too often official guidelines for practice and the latest evidence-based practice recommendation are not fully incorporated into clinical practice ‘on the ground’.[22,23] Knowledge gaps, skill deficiency, motivation, and system problems (supplies, infrastructure) all contribute to decreased quality of care.[5,9] However, research has shown that simulation and team-training can improve provider response to patient outcomes in general.[24,25]

Combined focus on the quality of care during physiologic birth and during obstetric and neonatal emergencies inside institutions through training and systems change has great potential.[1,9,16] Since the importance of the technical quality of care of normal deliveries has only been recently recognized, to our knowledge there are no reports in Mexico about strategies devised to improve it.

This report presents the results of a secondary analysis of a cluster-randomized trial, with the objective of estimating the impact of PRONTO, a simulation-based training intervention, on the performance of evidence based routine practices during delivery.

## Material and methods

### Study design

A pair-matched cluster randomized controlled trial of the intervention was designed to measure the impact of the program (PRONTO intervention) on a sample of 24 hospitals (12 intervention and 12 control) in the states of Chiapas, Guerrero, and Mexico State.[26] Data was collected at 4 time points (baseline, 4^th^, 8^th^ and 12^th^ months after the training). The units of analysis for this study were individual observed births (n = 641).

**Fig 1:**
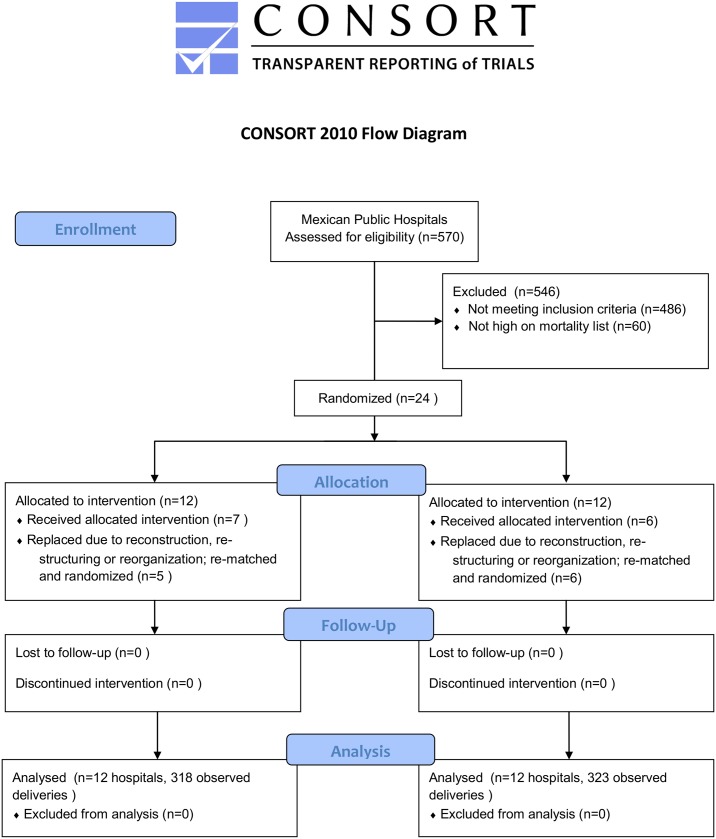
Flow chart for the selection of the sample.

The analytic sample was comprised of 641 observed births in twenty-four hospitals selected for the study: 318 in the treatment arm and 323 in the control arm (Fig 1). The first stage was to select hospitals that offer obstetric services of the Ministry of Health in Mexico (n = 570). As an inclusion criteria, the next step selected only level 2 hospitals, which had between 500 and 3000 annual deliveries (n = 265). This step was taken for logistic and financial considerations. Secretaries of Health of the states of Guerrero, Chiapas and Mexico were approached to secure permission to implement the training and the evaluation. There were initially 84 eligible hospitals of said states, of which 24 were selected for being in the high-mortality list. The sample size was determined to be sufficient to detect an impact on obstetric emergency indicators for the main objective of the study, as previously described (see Power calculation, below).[26] Prior to the start of baseline data collection, however, 11 of the 24 selected hospitals were replaced because they were unable to participate for a variety of reasons including restructuring, remodeling, or natural disaster damage; replacement was done by randomly selecting from among the remaining pool of 60 eligible hospitals not in the high-mortality list. Each pair of replaced hospitals was randomized to the intervention after the replacement occurred, by selecting the pair member with a higher value of a randomly generated uniform distribution with values between 0 and 1; these procedure was performed by the main analyst of the team at that time, who communicated the selected hospitals to the team of trainers (Fig 1). The main outcomes of the controlled trial were maternal obstetric complications and perinatal mortality; the impacts of the intervention on these primary outcomes along with more details on the selection process are described elsewhere.[26]

The 24 hospitals were pair-matched based on three variables: a) size of the hospital, measured through the number of births attended; b) number of obstetric complications by type; and, c) capacity, based on number of medical staff and available infrastructure.[26] After matching, a member of each matched-pair was randomly assigned to receive the intervention or to serve as control.

### Power calculation

The statistical power of the original study was calculated aiming to the its main objective, which was to estimate the impact of the intervention on rates of patient level outcomes such as neonatal death, obstetric hemorrhage, hysterectomies and eclampsia, among others (published elsewhere).[26] At the time of study planning it was estimated that the 12 matched hospital pairs would yield a total of 8400 births, and that this would be sufficient to achieve a power close to 60% to detect a 75% reduction in the outcomes (one-sided test), considering a difference-in-differences estimation of the outcomes’ incidence, with a significance level of 0.05, a design effect of 38.4 and an intra-class correlation of 0.05.

As power calculation for the main study was not done considering individual and direct birth observations, we performed a separate power analysis for the available sample of observed deliveries. We calculated that considering 12 clusters (hospitals) per arm, with an intra-class correlation of 0.05, and an average cluster size of 27 deliveries per hospital, the minimum detectable between-arms difference (two-sided test) in the probability of performing a practice at delivery, with a power of 80%, and a significance level of 0.05, ranged between 10 percentage points for DCC and 15 percentage points for uterine sweeping.

### Intervention

PRONTO (Spanish for *Programa de Rescate Obstétrico y Neonatal*: *el Tratamiento Óptimo y Oportuno*) is a simulation-based program that provides training for interprofessional teams (general physicians, nurses, specialists and students) using highly-realistic and low-cost simulation.[27] The PRONTO training in Mexico consists of 2 Modules: Module I includes: (a) a facilitated dialogue around humanized birth, patient communication and evidence based-practices (AMTSL, DCC, SSC, episiotomy, fundal pressure, uterine sweeping); (b) teamwork and communication; and (c) emergency management simulations and skills-stations focusing on obstetric hemorrhage and neonatal resuscitation). Module II (2 to 3 months after Module I) reinforces Module I themes and adds shoulder dystocia and preeclampsia/eclampsia. PRONTO trainings utilize the hybrid birth simulator PartoPants with a patient actress (often a hospital staff/training participant) to simulate birth and the Laerdal NeoNatalie for the neonatal resuscitation.^27^ Simulation scenarios, when possible, occur in the actual clinical setting (emergency department, labor ward, delivery room, operating room, recovery room) where care is provided. Team training includes activities that reinforced concepts of leadership, and communication techniques adapted from the TeamSTEPPS Program.[24]

Twelve hospitals in the intervention arm of the study received PRONTO between August 2010 and January 2012. The selection of trainees was discretionally decided by the health facilities’ authorities; with only a strong recommendation from the program that trainees attended deliveries or worked in emergency or delivery rooms.

For each of the 12 intervention hospitals the total personnel was between 90 and 467, for the smallest and largest hospitals respectively.[28] Between 6% and 32% of the total personnel per hospital received the Module I training and between 4% and 25% for Module II (mean = 21%), of all trainees 54% where physicians and 46% were nurses. Details on trainee characteristic and the PRONTO training are reported elsewhere.[28]

### Data collection

Five trained observers collected information on hospital infrastructure, equipment, services and human resources. The goal was to observe at least 10 vaginal births by different providers on different shifts in each hospital over a maximum 5-day period at each visit. Prior to birth observation, both the health provider and the laboring woman provided oral consent. Observers were instructed to watch deliveries starting from the second stage of labor and ending 10–20 minutes after the third stage was completed. The observers used a checklist to collect data and were not blinded to treatment allocation. Data collection began in August 2010 and follow-up concluded in March 2013.

### Variable definitions

The main outcomes were the performance of the following routine practices: 1) AMTSL defined as: (a) applying 10 international units of oxytocin in the first minute after the birth of the baby, (b) traction and counter-traction of the umbilical cord, and (c) uterine massage immediately after the birth of the placenta. 2) Use of DCC, defined as a delay in the clamping of the umbilical cord of at least 60 seconds after the birth of the baby. 3) SSC defined as immediate contact between mother and child after birth. 4) Episiotomy defined as the performance of an incision in the female perineum including skin, muscular plane and vaginal mucosa. 5) Fundal uterine pressure, defined as application of manual pressure on the upper part of the uterus directed towards the birth canal. 6) Uterine sweeping, defined as the introduction of the hand or clamps into the uterus after the birth of the placenta.

The intervention variable was the hospital assignment to the training (yes/no). Important covariates were health providers’ profession, availability of medications, facility infrastructure (services provided) and incidence of obstetric complications.

### Analysis

The outcome variables were defined as a dummy variable for the performance of the routine practice at each delivery (0 = no, 1 = yes). For bivariate treatment group comparisons of the outcome variables at baseline, simple logistic regression models considering robust standard errors were fitted to the data. To estimate the impact of the PRONTO training on the probability of performing a given practice at 4, 8 and 12 month post intervention, we fitted difference-in-differences [29] mixed effects logistic regression models. All models considered fixed-effects of the matched pairs to control for this aspect of the study design and inclusion of the attending provider’s profession (intern, resident, general practitioner, obstetrician, nurse or other) as a covariate. After fitting the models, impact estimates were expressed in terms of the change in the probability of the outcome. All analyses were performed using STATA v. 13.0 (StataCorp. 2013. College Station, TX: StataCorp LP).

### Ethics

We obtained verbal consent from all women whose deliveries were observed, as well as from the health care providers who attended those deliveries. Study staff was instructed to abandon the delivery room in case consent wasn’t given or in case a clear answer was not obtained. Consent was verbal as it was deemed impractical and intrusive to ask for written concern in the midst of labor/delivery; this procedure along with all instruments for data collection and for pre- and post-training evaluations, letters of consents, as well as the study protocol were approved by the Ethics and Research Committees of the National Institute of Public Health in Mexico (Reference 845, August 2, 2010). The trial is also registered at clinicaltrials.gov: NCT01477554; registration was done after enrollment began as the research team was unsure at the time on whether trials such as this (with no direct intervention on patients) were eligible for registration. The authors confirm that all ongoing and related trials for this intervention are registered

## Results

Baseline analyses results for hospital infrastructure, routine practices and other related variables in studied hospitals are presented in Tables 1 and 2. For all except two (Neonatal Intensive Care Unit [p = 0.019] and number of general practitioners [p = 0.026]) of 48 variables tested, the means or proportions of control and intervention hospitals were not statistically different at baseline.

**Table 1:** Hospital infrastructure and personnel characteristics of the 24 participant facilities a bivariate comparisons performed by linear regression models with clustering at the matched pair level and robust standard errors.

Variables	Baseline		
	Control n = 12	Treatment n = 12	p-value^a^
**Health professionals**	Mean (SD)	Mean (S.D)	
Total doctors	79.4 (33.8)	70.1 (49.1)	0.416
Total nurses	155.3 (78.5)	133.9 (62.8)	0.262
General Practitioners	39.0 (21.3)	28.8 (15.0)	0.026
Obstetricians	10.4 (5.09)	9.7 (6.0)	0.699
Anesthesiologists	9.3 (4.1)	8.0 (4.7)	0.419
Pediatricians	9.4 (4.5)	7.3 (6.3)	0.191
Surgeons	5.8 (3.9)	5.6 (3.9)	0.873
Neonatologists	1.1 (2.4)	1.2 (2.3)	0.881
Internists	3.0 (2.3)	3.2 (4.4)	0.878
Nurses	150.2 (76.7)	127.8 (59.0)	0.232
Obstetric nurses	5.2 (5.8)	8.1 (7.5)	0374
Midwifes	0.0 (0.0)	0.4 (1.4)	0.339
**Facility Infrastructure**	%	%	
Blood transfusion capability	50	58	0.594
Neonatal Intensive Care Unit	83	42	0.019*
Adult Intensive Care Unit	50	25	0.200
Obstetric hemorrhage algorithm	45	58	0.584
Preeclampsia/ eclampsia algorithm	58	67	0.681
Neonatal resuscitation algorithm	55	45	0.668
Official Mexican Law for birth attendance (NOM007)	58	64	0.834
Obstetric emergencies manual	75	55	0.380
Obstetric emergency triage system	50	50	1
Number of referral hospitals	3.71	4	0.822
Distance to most used referral hospital (Km)	54.17	89.33	0.351
**Facility Equipment**	%	%	
Ambulance	75	92	0.349
Laboratory	100	100	1
Ultrasound	100	100	1
Doppler	64	67	0.891
Uterine manual vacuum aspiration (AMEU)	50	75	0.200

^a^ Bivariate comparisons performed by linear regression models with clustering at the matched pair level and robust standard errors.

* p-value <0.05

**Table 2:** Medication, services offered and birth practices at baseline in the 24 participant facilities.

Variables	Baseline		
	Control n = 12	Treatment n = 12	p-value^a^
**Facility Medications**	%	%	
Oxytocin	100	92	0.349
Misoprostol	50	25	0.285
Antibiotics (ampiciline)	100	100	1
Antihypertensive (hidralazine)	83	100	0.175
Magnesium Sulfate	83	82	0.937
**Services**^b^	Mean (SD)	Mean (SD)	
Labor and Delivery Room	1.1 (0.29)	1.3 (0.45)	0.175
Operating Rooms	1.3 (0.45)	1.2 (0.39)	0.594
Births	573.1 (386.0)	474.5 (198.3)	0.477
Cesareans	263.1 (232.0)	235.5 (125.4)	0.743
Abortions	88.0 (63.9)	66.1 (36.7)	0.242
Curettage	96.9 (75.0)	71.4 (56.5)	0.338
Obstetric hemorrhage	8.2 (10.1)	19.3 (32.1)	0.341
Hysterectomy	3.9 (4.0)	2.2 (2.1)	0.164
**Delivery Practices at baseline**^c^	**n = 88 (%)**	**n = 58 (%)**	**p-value**^c^
Complete Active Management of the 3rd Stage of Labor (AMTSL)^d^	17.0	18.2	0.927
First Step of AMTSL	27.3	32.1	0.747
Skin-to-Skin Contact (SSC)	6.80	11.3	0.579
Delayed Umbilical Cord Clamping (DCC)	14.92	11.9	0.714
Episiotomy	64.3	65.4	0.911
Fundal Pressure (Kristeller maneuver)	20.0	13.4	0.370
Uterine Sweeping	80.6	85.2	0.635

^a^ Bivariate comparisons performed by linear regression models with clustering at the matched pair level and robust standard errors.

^b^ Data collection in this section considering 3 previous months.

^c^ Bivariate comparisons performed by logistic regression models with robust standard errors and clustering at the hospital level.

^d^ AMTSL consisting of 3 steps: applying 10 units of oxytocin in the 1st minute after the birth, traction and counter-traction of the umbilical cord and uterine massage immediately after the birth of the placenta.

A total of 641 births were observed over the study period with an average of 6.25 births (SD 2.8, range 1–12) per visit to each hospital; 318 of these occurred in treated hospitals. Performance of birth practices at baseline also showed balance (Table 2).

**Fig 2:**
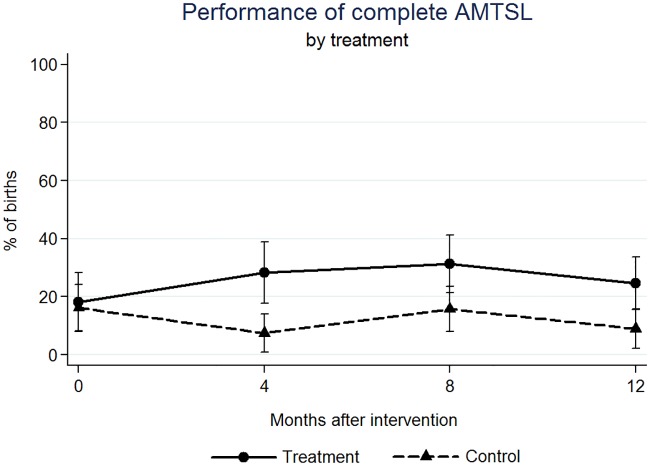
Estimated percentage of births in which a complete Active Management of the Third Step of Labor was performed, by treatment (PRONTO training) group.

**Fig 3:**
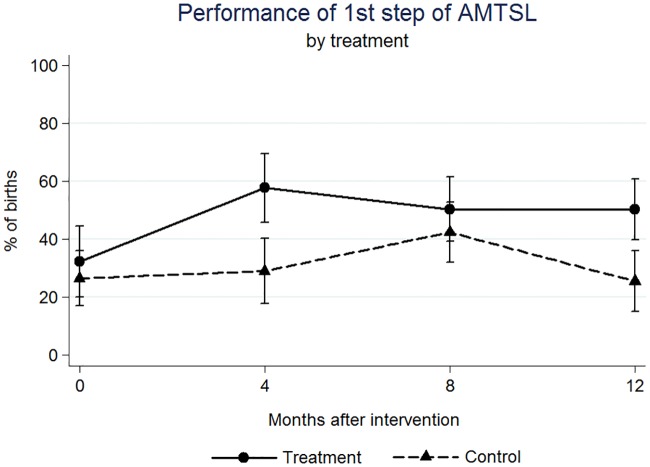
Estimated percentage of births in which the 1^st^ step of the Active Management of the Third Step of Labor was performed, by treatment (PRONTO training) group.

**Fig 4:**
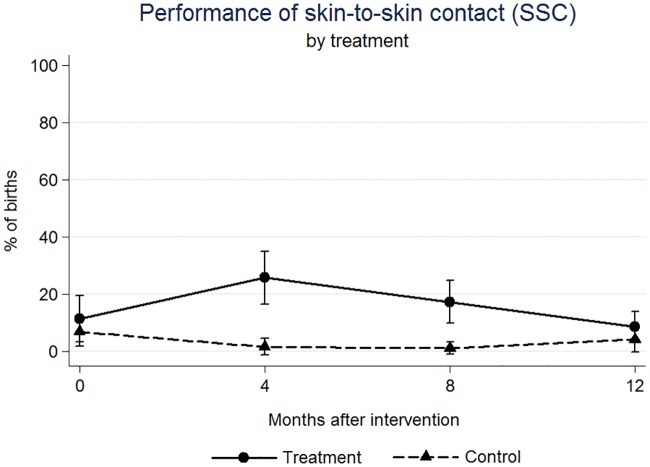
Estimated percentage of births in which Skin-to-Skin Contact was performed, by treatment (PRONTO training) group.

**Fig 5:**
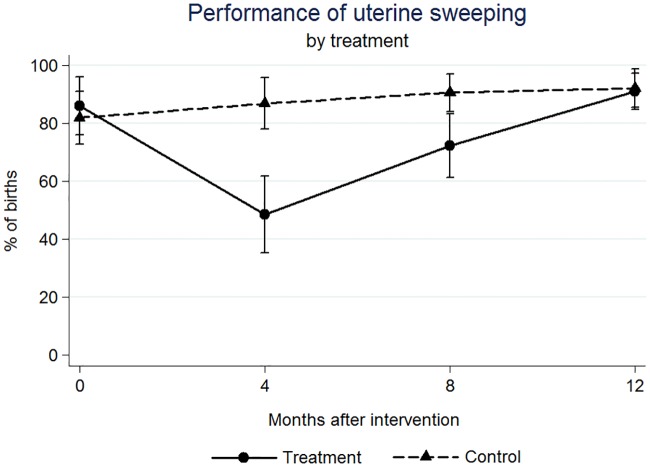
Estimated percentage of births in which Uterine Sweeping was performed, by treatment (PRONTO training) group.

We found significant effects on the outcome variables at four, eight, and twelve months after the intervention (Figs 1–5). At four months post intervention we estimated an increase of 20 percentage points in the probability of performing complete AMTSL practice (p-value = 0.044) (Fig 2) and a 16 percentage point increase for SSC (p-value = 0.067) (Fig 4). At four months we estimated a 21 percentage point increase (p-value = 0.070) and at twelve months a 25 percentage point increase in the performance of the 1st step of AMTSL (p-value = 0.026) (Fig 3), and a 42 percentage point increase of DCC (p-value = 0.004). At eight months we estimated an 18 percentage point increase of fundal pressure (p-value = 0.034) and at four and eight months a 30 (p-value = 0.001) and 22 (p-value = 0.010) percentage point decrease of uterine sweeping, respectively (Fig 5). The apparent increase of fundal pressure and DCC are mainly driven by an unexplained decrease of the incidence of these practices in the control group at eight (3% [95% CI: 0, 6.9%] vs. 27% [95% CI: 17, 37%] at four months) and twelve months (61% [95% CI: 47, 75%] vs. 85% [95% CI: 72, 98%] at eight months) post intervention, respectively. The inclusion of a variable for other courses directed at obstetric training did not noticeably change the impact estimates, nor did including a variable to adjust for observer. The complete set of impact estimates appears in Table 3.

**Table 3:** Estimated impact of the intervention (PRONTO training) over the probability of routine practices at birth^a^.

Variables	4 month post intervention		8 month post intervention		12 month post intervention	
	Impact^c^	p-value	Impact^c^	p-value	Impact^c^	p-value
Complete AMTSL^b^	0.203	0.044**	0.099	0.240	0.141	0.133
1st step of AMTSL^b^	0.211	0.070*	0.082	0.444	0.249	0.026**
Skin to skin contact (SSC)	0.164	0.067*	0.129	0.149	-0.022	0.752
Delayed cord clamping (DCC)	-0.140	0.287	0.046	0.696	0.419	0.004**
Episiotomy	-0.058	0.612	-0.127	0.238	-0.097	0.386
Fundal pressure (Kristeller maneuver)	-0.079	0.265	0.175	0.034**	0.036	0.622
Uterine sweeping	-0.296	0.001**	-0.223	0.010**	-0.039	0.676

^a^ Impact estimates were obtained by fitting difference-in-differences mixed-effects logistic regression models adjusting for provider´s profession. The matched structure of the data was considered including dummy variables of the matched pair. Afterwards, we obtained marginal effects for interpretation in terms of the change in the probability of the outcome.

^b^ Active Management of Third Stage of Labor

^c^ Change in probability of the outcome

** p-value <0.05;

* p-value <0.10

## Discussion

The performance of evidence-based practices during physiologic birth was found to be similar to previous studies in Mexico.[14–16]

Although we found significant impacts, most changes were neither consistent nor sustained over time. Only two evidence-based practices were found to be significantly affected by the training at twelve months post intervention: first step of AMTSL and DCC. Also we found an apparent increase of fundal pressure at eight months driven by an unexplained decrease of the incidence of this practice in the control group which might be due to problems in the assessment of this practice by the observers or by other training efforts we were unaware of, as well as any changes in the personnel at control hospitals who did not practice fundal pressure.

It is important to point out that the training primarily focused on emergency obstetric and neonatal care; the session on normal birth practices was a relatively short discussion (about 45–60 minutes) during Module I, and the concepts were reviewed in three scenarios and their respectively simulations’ debriefs (of a total of six simulations and 16 hours of training). In light of this relatively minor focus, it is interesting to see that normal birth practices were significantly impacted. This suggests that a training specifically geared towards normal physiologic birth may have a larger, more lasting impact. The use of the simulator in which one of the trainees played the role of the patient may have also contributed to a behavioral change even in the most entrenched practices. In other words: having the nurse or doctor in the simulations reflect on the experience of being a “patient” may have helped to change their behavior. More research should be done to look into the power of this phenomenon.

PRONTO combines training in emergency response with normal birth care within the context of inter-professional team training and grounded in highly-realistic in-situ simulation. This type of learning is new in Mexico, where medical training emphasizes traditional authority-based medicine and entrenched clinical practices with lesser push to institute evidence-based care. This phenomenon may reflect a lack of current knowledge or access to recent research and/or a lack of incentives coupled with a disincentive (punishment) for the use of evidence-based practices that contradict leadership.[9,15] Another limitation of medical training in Mexico is a lack of repetitive practice inside the hospitals. The training strategy used during the intervention may have filled these gaps in the treated hospitals.

Although we found significant changes for the main outcomes at different follow-up periods, we also found a dilution of the effect over time. There are several possible explanations that could account for this. First, we could not determine if we were training innovators and early adopters [30] or those so entrenched in their beliefs, that no training would change their behavior. Furthermore, there is a high rate of staff turnover, especially within the nursing staff which rotates between services and new interns arriving every 6 and 12 months. Finally, there is evidence that effective training efforts require reinforcement mechanisms and top-off trainings because learning might last 6 months [31] which, although PRONTO teams returned for a second module three months after the first, there was no follow-up after this.

Large fluctuations in the incidence of some practices such as fundal pressure and DCC at different time-points, particularly in the control group, may reflect a lack of clarity of these concepts by the observers. However, observers were not assigned to a particular hospital and they were indistinctly sent to either a control or treated unit at any given point in time. Additionally, even though observers were not blinded to the treatment assignment of the facility, they were unable to assess which of the observed providers had participated in the training and which had not, and more importantly, they were instructed not to discuss training participation with providers, which leads to greater confidence in the results. In fact, an additional set of models adjusting for observer yielded the same results (not shown).

Another important limitation is that we did not collect variables on the level of the individual mother whose birth was observed. Some of these non-observable variables such as the mothers’ education or socio-economic level may influence health professionals’ provision of care.[32] However, since the intervention was randomized, we expect the distribution of these covariates to be balanced between intervention and control groups. Additionally any remaining time-invariant confounders that failed to be equally distributed between arms by means of the matching and the randomization, were likely controlled by the Difference-in-Differences approach, [27] leaving little room for bias. A final important limitation is that this study was conducted in very particular settings within Mexico, which may yield these results not generalizable to other settings in the country or abroad.

A major strength of this study was the design and randomization of the larger intervention. The methodology used, including the analytical strategy, was devised to correctly estimate the impact of this program, even considering the implementation problems. We need to conduct additional analyses to estimate the actual impact on birth attendance practices specifically in trained personnel, isolating it from the effect on those not trained in the intervention hospitals (average treatment-on-the-treated effect or ATT). This is due to the fact that only an average of 21% of total eligible personnel was trained in the 12 interventions hospitals. The budget constraints and timeline of the project was a limitation for training a greater proportion of the personnel and thus the designers’ expectation was that trained participants would share their new knowledge to those who were not trained. Since we found a dilution of the effect, for future interventions we recommend to train a proportion of eligible personnel that is closer to a 100% and to have continuing education events or refreshers at least once a year to increase up-take and reinforce behavioral changes.

## Conclusions

PRONTO has an impact on evidence-based routine practices at birth, contributing to an increased quality of care as categorized by recent frameworks.[1] The potential to have a long lasting impact on the performance of evidence-based practices would be greater with widespread adoption of the training strategy including simulation, team-training and facilitated discussions regarding adequate care. Additional research should include a more in- depth analysis of the dilution of the effect over time.

## Supporting information

S1 CONSORT Checklist CONSORT checklist. (PDF)

S1 Protocol Study protocol in Spanish. (PDF)

S2 Protocol Study protocol in English. (PDF)

S1 Dataset Hospital level dataset. (XLSX)

S2 Dataset Delivery level dataset. (XLSX)
