# Supplementary material for: Can a simulation-based training program impact the use of evidence based routine practices at birth? Results of a hospital-based cluster randomized trial in Mexico
Source: PLoS One. 2017 Mar 20;12(3):e0172623. doi: 10.1371/journal.pone.0172623 (PMC5358738; doi:10.1371/journal.pone.0172623)
Supplement: S1 Protocol — (PDF) [file pone.0172623.s002.pdf]

PROTOCOLO DEL PROYECTO DE INVESTIGACIÓN:

PRONTO "Programa de Rescate Obstétrico y Neonatal: Tratamiento Óptimo y Oportuno. Ensayo aleatorizado para una evaluación de impacto"

Responsable del proyecto:

Dilys Walker, MD

Associate Professor University of Washington Depts. of Ob Gyn and Global Health

Equipo:

Dra. Sandra Gabriela Sosa Rubí, INSP

Dr. Martín Romero, INSP

CNM Susanna Cohen, University of Utah School of Nursing

CNM, MPH Jennifer Fahey, University of Maryland School of Medicine

Mtra. Dolores González Hernández, INSP

RESUMEN

Las tasas y razones de mortalidad materna en México han tenido un descenso ligero, por lo que cumplir con las metas de desarrollo del milenio (MDM) comprometidas internacionalmente por nuestro país es una meta lejana. La atención adecuada y oportuna de las emergencias obstétricas es un elemento fundamental para la reducción de la mortalidad materno-infantil. PRONTO^2: Programa de Rescate Obstétrico y Neonatal: el Tratamiento Óptimo y Oportuno, es una estrategia de capacitación innovadora basada en simulaciones, para la capacitación de personal de salud en hospitales para la atención de emergencias obstétricas. El objetivo de este estudio es implementar la intervención PRONTO^2 en hospitales seleccionados de manera aleatoria, para medir la efectividad de la intervención en comportamientos clave de la práctica hospitalaria en la atención de emergencias obstétricas (indicadores de proceso), así como recolectar información sobre indicadores de resultado en hospitales de intervención y control para realizar una evaluación de impacto del programa PRONTO^2.

INTRODUCCIÓN

Las tasas y razones de mortalidad materna en México han tenido un descenso ligero, por lo que cumplir con las metas de desarrollo del milenio (MDM) comprometidas internacionalmente por nuestro país es una meta lejana. PRONTO^2: Programa de Rescate Obstétrico y Neonatal: el Tratamiento Óptimo y Oportuno, es una estrategia innovadora basada en simulaciones, para la capacitación de personal adscrito a los servicios de salud en hospitales comunitarios y de referencia para la atención de emergencias obstétricas.

Numerosas evaluaciones sobre mortalidad materna han indicado que la calidad de los servicios obstétricos así como el otorgamiento de la atención oportuna y adecuada de las emergencias obstétricas es clave para disminuir las tasas de morbi-mortalidad materna en el ambiente hospitalario. Los modelos de capacitación tradicional, como sesiones didácticas o la introducción de guías y protocolos no han mostrado los resultados esperados en los indicadores de interés. PRONTO^2 utiliza una metodología única en su tipo en México, con un modelo de simulaciones (con alta fidelidad y baja tecnología), para capacitar equipos multidisciplinarios encargados de atender las emergencias obstétricas hospitalarias, para lograr la atención de las emergencias de una manera efectiva y exitosa incorporando de manera integral elementos de conocimientos, prácticas, disponibilidad de insumos e infraestructura hospitalaria, así como los sistemas de salud.

Durante el año 2009 el equipo de trabajo del programa PRONTO^2 (Programa de Rescate Obstétrico y Neonatal: el Tratamiento Óptimo y Oportuno) desarrolló el currículo, los manuales de implementación y realizó un estudio piloto en 2 hospitales del estado de México y en 2 hospitales en el estados de Chiapas, que fueron seleccionados por los Institutos de Salud de cada uno de los estados. El estudio piloto consistió en la implementación del currículo de PRONTO^2 para personal del hospital, cubriendo 15 horas de capacitación. Al finalizar la implementación se hizo un reporte, que fue entregado a cada uno de los hospitales, en el cual se puntualizaban metas específicas y factibles a cumplir, que fueron establecidas por los participantes para mejorar la atención de emergencias obstétricas dentro de sus hospitales. Los resultados preliminares de este piloto, así como el seguimiento a los 3 meses de la implementación, ha mostrado no solo

la factibilidad de llevar a cabo la intervención PRONTO[2] in situ, sino que quedó registrada la aceptabilidad por parte de los Secretarios de Salud, los directivos de los hospitales, así como por los participantes tanto de la metodología de aprendizaje a través de las simulaciones, como de las modificaciones observadas en los participantes a raíz de la implementación PRONTO[2]. Por tanto, el siguiente paso es llevar a cabo una implementación aleatorizada y controlada para evaluar de una manera rigurosa el programa, para determinar el impacto que tiene PRONTO[2] en las prácticas de la atención de emergencias obstétricas y en resultados obstétricos y neonatales de corto y largo plazo, así como en el trabajo de equipo, comunicación entre los miembros del equipo médico y con la paciente.

ANTECEDENTES

La mortalidad materna constituye un importante problema de salud pública en nuestro país. Si bien en los últimos años se ha documentado un descenso en la mortalidad materna, pasando de acuerdo a estadísticas oficiales de 89 muertes por cada 100,000 nacidos vivos en 1990 a 63.4 en el año 2005; siguen existiendo regiones del país donde las tazas aún siguen altas, sobre todo en estados como Guerrero, Oaxaca y Chiapas.[1,2]

La mortalidad materna constituye un buen indicador de las condiciones sociales y económicas de las mujeres.[3] Aunque la frecuencia de las defunciones maternas en números absolutos es baja comparada con otros problemas de salud pública, detrás de la muerte de una mujer existen graves consecuencias como la desintegración de la familia, una elevada mortalidad y morbilidad infantil, deserción escolar, desnutrición, orfandad y el ingreso prematuro de los hijos al mercado de trabajo.[4] Ante esta problemática, el sector de salud ha instrumentado diversas acciones a distintos niveles para reducir la mortalidad materna. La reducción de la mortalidad materna es una de las Metas de Desarrollo del Milenio (MDM) suscritos por nuestro país. Específicamente, se ha propuesto la meta cuatro de reducir, entre 1990 y 2015, la mortalidad materna en tres cuartas partes.[5] Cuando estábamos a 10 años de concluir el tiempo establecido en las MDM, el avance era de menos de 45%.[1] La mortalidad materna es la 4ª causa de muerte en mujeres en

edad reproductiva en México y ocurre principalmente por causas relacionadas con emergencias obstétricas: 25% la enfermedad hipertensiva del embarazo y 24% por hemorragias durante el embarazo y el parto.[1]

Asimismo, la cuarta meta del milenio propone una reducción en la mortalidad infantil de dos tercios, entre 1990 y 2015, actualmente México se encuentra cinco puntos porcentuales debajo de la meta.[6] En México, más de la mitad de las muertes en niños menores de 5 años, son perinatales y ocurren en las primeras 24 horas de vida. El llevar a cabo intervenciones hospitalarias enfocadas en la reanimación del recién nacido en el primer minuto de vida, son efectivas para reducir la morbi-mortalidad neonatal.[7,8]
De acuerdo a datos del 2004, 12 entidades federativas concentran el 75% del total de las defunciones maternas: Chiapas, Guerrero, Oaxaca, Puebla, Durango, Veracruz, Hidalgo, el Estado de México, San Luis Potosí, Baja California, el Distrito Federal y Querétaro.[2]
A diferencia de la situación en años anteriores (al menos hasta la década de los 90), una alta proporción de muertes maternas ocurren en unidades hospitalarias.[9] Una análisis reciente de datos del INEGI (2005) encontró que en casi 3/4 partes de los municipios del país la proporción de los partos atendidos en unidades médicas es superior al 70%.[10]

Estrategias efectivas para combatir la morbi-mortalidad materna.

Se han desarrollado y evaluado diferentes estrategias para incidir en la disminución de la mortalidad materna y perinatal. Dado que es un problema complejo, no existe una estrategia universal para combatir o reducir las tazas de mortalidad materna y perinatal. Algunas de las estrategias relevantes se mencionan a continuación:

1. **Mejorar la calidad de la atención médica para mujeres embarazadas, sobre todo cuando se enfrenta una emergencia obstétrica.** Un elemento esencial para la reducción de la mortalidad materna es otorgar servicios de salud de calidad durante el embarazo y al nacimiento.

Una buena proporción de muertes maternas ocurren al momento del nacimiento, con diagnósticos de hemorragia obstétrica y preeclampsia/eclampsia, por lo que el brindar atención oportuna y adecuada de las emergencias obstétricas es la clave para bajar las tasas de mortalidad materna en el ambiente hospitalario. Frecuentemente estas

complicaciones no se presentan hasta el momento de trabajo de parto o nacimiento. En general aproximadamente 10% de los partos van a complicarse por una razón u otra.[11] Igualmente hay estimaciones en que la atención durante el parto y en las primeras horas de vida del recién nacido son críticas para su sobrevivencia.

Numerosas evaluaciones sobre mortalidad materna han indicado que la calidad de los servicios obstétricos es un asunto importante que debe atenderse. La mejora sistemática de estos servicios en países en desarrollo como Malasia y Sri Lanka, han logrado abatir las tasas de mortalidad materna hasta en un 50% en un periodo de 7 a 10 años.[12]

2. Fortalecer la infraestructura de las unidades médicas. Es posible ofrecer servicios obstétricos de calidad en las comunidades pero es necesario fortalecer la infrastructura necesaria.[13] Existen ejemplos de hospitales regionales en Rwanda, África en donde la renovación de los hospitales junto con su equipamiento y capacitación del personal, dieron como resultado un hospital capacitado para atender emergencias obstétricas con menos complicaciones, muertes materno-infantiles, sin alterar sus cifras de cesáreas.[14] En Latinoamérica existen ejemplos como Perú, donde la mejora en instalaciones y los servicios para la atención de emergencias obstétricas dieron resultados positivos en la reducción de la mortalidad materna.[15]

3. Auditorías periódicas con retroalimentación para medir calidad de atención. Para evaluar la calidad de atención, se han implementado desde hace varios años auditorías, las cuales se definen como el análisis crítico sistemático de la calidad de la atención médica e incluye los procedimientos de diagnóstico, el tratamiento, el uso de los recursos así como la calidad de vida del paciente.[16] Se considera que las auditorías con retroalimentación de los servicios, pueden ayudar a mejorar la utilización de recursos y la calidad de la atención de emergencias obstétricas, especialmente en los países con recursos limitados.[17] Para lograr la implementación de las auditorías clínicas las autoridades de los servicios de salud deben invertir en capacitación y monitoreo de sus servicios médicos.[18]

4. Capacitación con técnicas nuevas como simulaciones. En estudios previos realizados del 2004 al 2007 por el grupo de investigación liderado por la Dra. Dilys Walker, han evaluado la calidad de la atención ofrecida por distintos proveedores

vinculados con la atención obstétrica: médicos generales, enfermeras obstetras y parteras profesionales técnicas. En este contexto se desarrolló una estrategia para capacitar a estos proveedores en la atención de emergencias obstétricas, utilizando simulaciones en prácticas clínicas basadas en evidencia científica y en deficiencias detectadas en las evaluaciones previas que estos proveedores tuvieron en su práctica obstétrica. Una evaluación de este capacitación, mostró que el conocimientos y la auto-eficacia aumentó de una manera significativa y se mantuvo hasta 3 meses después de la capacitación.[19] Nuestra experiencia para capacitar a personal de salud vinculado con la atención del parto, mediante el uso de simulaciones junto con nuestras experiencias más reciente en el 2009, con la implementación de PRONTO^2 en cuatro hospitales, nos da el soporte técnico y la experiencia para desarrollar esta nueva fase de investigación que contempla el desarrollo de un estudio de investigacion denominado **PRONTO^2 Programa de Rescate Obstétrico y Neonatal: Tratamiento Óptimo y Oportuno: Ensayo aleatorizado para una evaluación de impacto,** que estará dirigido a implementar la intervención en una muestra de hospitales seleccionados aleatoriamente (descrito abajo) para capacitar a un grupo interdisciplinario de personal adscrito a los hospitales seleccionados de la SSA, vinculados con la atención de emergencias obstétricas en hospitales de segundo y tercer nivel de atención.

PRONTO^2 utilizará una técnica de aprendizaje basada en el uso de simulaciones para el manejo de emergencias obstétricas en el ambiente hospitalario, con esta estrategia se busca mejorar la calidad de atención ofrecida en caso de una emergencia obstétrica para lo cual se obtendrá información en tres momentos de resultados obstétricos y neonatales, así como de proceso para realizar una evaluación de impacto del programa PRONTO^2.

JUSTIFICACIÓN

La atención adecuada y oportuna de las emergencias obstétricas es un elemento fundamental para la reducción de la mortalidad materno-infantil.[20] Dado que la mayoría de las muertes maternas en México ocurren en el ambiente hospitalario y hay evidencia de que la calidad de la atención ofrecida es un elemento importante en las causas de muerte, es crítico desarrollar y evaluar estrategias novedosas para aumentar la calidad de la atención en el caso de las emergencias obstétricas. En este sentido proponemos una

estrategia basada en el uso de simulaciones en el sitio en donde están ocurriendo las emergencias para intentar mejorar la calidad de atención. Hasta el momento los métodos tradicionales de aprendizaje como cursos didácticos, folletos, guías y manuales no han mostrado tener una alta efectividad en el cambio de comportamiento de proveedores en el uso de prácticas basadas en evidencia.[21]

La frecuencia con que ocurren las emergencias obstétricas es baja, por lo que el personal de salud no tiene práctica clínica repetitiva que le permita integrar sus experiencias, conocimientos y habilidades al campo práctico. El método de aprendizaje a través de simulaciones, ofrece la posibilidad de participar en la recreación de un caso clínico real en donde a través de la experiencia vivencial entre el equipo médico sea posible identificar cómo deben actuar, interactuar y resolver de manera satisfactoria una emergencia obstétrica. La simulación es un sistema de enseñanza adaptado de la industria de aviación espacial a la medicina y tiene como objetivo incrementar el conocimiento, mejorar las habilidades, para formar liderazgo y asegurar la movilización eficiente de recursos y la coordinación apropiada de la atención a la salud.[22]

La simulación se define como un entrenamiento que pretende la inmersión del estudiante en una situación real (escenario), creada dentro de un espacio físico (simulador), que replica el entorno real con la suficiente fidelidad para conseguir que el estudiante lo crea verdadero. Parte de la metodología de la simulación incluye la filmación de toda la simulación para garantizar la reproducción exacta del comportamiento del equipo en una situación de emergencia. A partir de su reproducción en un monitor frente a todo el personal participante, se realiza una retroalimentación inmediata lo que permite observar y evaluar la respuesta del equipo en entrenamiento, la coordinación con la que le ofrecen los cuidados de salud a la mujer y las habilidades de liderazgo que espontáneamente se manifiestan.

La evaluación de las estrategias implementadas en varios ambientes y en varios países para mejorar la atención a la salud, han identificado factores que anteriormente no se habían tomado en cuenta. Un error frecuente en los servicios de salud, que compromete la seguridad de la paciente, está ligado a fallas latentes en la estructura y función de los sistemas. El cuidado de las pacientes, está a cargo de un equipo integral de prestadores

de servicios de salud por lo que el reto actual para los programas de capacitación de los profesionales de la salud está en incrementar la experiencia del trabajo en equipos multidisciplinarios. La complejidad de capacitación de estos equipos requiere de sistemas multifuncionales que van más allá de las divisiones organizacionales, para permitir la comunicación, la rendición de cuentas y el mantenimiento mismo del equipo.[23]

En la actualidad el entrenamiento de trabajo en equipo es crucial, especialmente dentro de los espacios de atención a la salud. PRONTO[2] incorpora elementos de un programa conocido como Team STEPPS, que dirige su atención a resolver los problemas de comunicación y fragmentación del trabajo. La capacitación y evaluación está centrada en el funcionamiento del equipo, para lograr la seguridad de la paciente.[24]

Debido a que las reglas para evaluar la mejoría de los servicios de atención de emergencias obstétricas aún están en estudio, la metodología del presente estudio, propone determinar junto con el hospital en estudio, los problemas de la práctica clínica que podrían necesitar solución, determinar los estándares de atención de ese problema para que se vigilen las prácticas y se mejoren.[25]

La obtención de indicadores a corto y largo plazo se basan en consideraciones prácticas. La selección de los indicadores a corto y largo plazo se selecionaran en función de los problemas a resolver. Unos ejemplos serían la disponibilidad de sangre en los casos de hemorragia posparto, la disponibilidad y uso de la oxitocina en el manejo activo de la tercera etapa del parto, la disponibilidad y uso del sulfato de magnesio en casos de preeclampsia severa, disminuir tasas de cesárea e histerectomía.

OBJETIVO GENERAL

El objetivo de este estudio es implementar la intervención PRONTO[2] en hospitales seleccionados de manera aleatoria, y medir la efectividad de la intervención en comportamientos clave de la práctica hospitalaria en la atención de emergencias obstétricas (indicadores de proceso), así como recolectar información sobre indicadores de resultado obstétricos y neonatales en hospitales de intervención y control para realizar una evaluación de impacto del programa PRONTO[2].

OBJETIVOS ESPECÍFICOS

- Medir el efecto del programa PRONTO^2 en indicadores de proceso.
- Medir el impacto del programa PRONTO^2 en indicadores de resultados obstétricos y neonatales

Preguntas de investigación:

1. ¿La capacitación PRONTO^2 mejora el trabajo en equipo del personal del hospital que atiende emergencias obstétricas?
2. ¿La capacitación PRONTO^2 mejora la comunicación entre los miembros del equipo que atiende emergencias obstétricas?
3. ¿La capacitación PRONTO^2 mejora la comunicación de los miembros del equipo que atiende emergencias obstétricas con la paciente?
4. ¿PRONTO^2 incrementa y mantiene a lo largo del tiempo los niveles en la escala de autoeficacia?
5. ¿Con la capacitación PRONTO^2 se observan cambios en la estructura y recursos del hospital? Por ejemplo:

    a. El lugar donde se almacena el equipo (ultrasonido, Doppler, refrigerador) y medicamentos (oxitocina, ergonovina), así como en las personas que tienen acceso a los mismos.

    b. Acceso visible a protocolos de manejo de emergencias obstétricas y neonatales (algoritmos de reanimación neonatal, manejo activo de la tercera etapa del parto (MATEP), uso de medicamentos para hemorragia obstétrica)

    c. Acceso a otros recursos como:

        i. Misoprostol
        ii. Carbetocina

iii. Sonda Foley
iv. Hemo cel

6. ¿Con la capacitación PRONTO^2 se observan cambios en las rutinas y prácticas del hospital?

    a. Implementar manejo activo de la tercera etapa del parto (MATEP)
    b. Evitar posición supina durante el parto
    c. Implementar pinzamiento tardío del cordón
    d. Medida preventiva para presencia de líquido meconial (aspiración inmediata, retrasar la estimulación)
    e. Contacto madre-neonato inmediato
    f. Aspiración manual endo-uterina (AMEU) para tratamiento de aborto en el primer trimestre
    g. Evitar limpieza uterina
    h. Evitar presión fundal (Kresteller)
    i. Disminuir episiotomías

7. ¿Con la capacitación PRONTO^2 se observan cambios en indicadores de resultados obstétricos y neonatales e indicadores epidemiológicos? Seleccionado de los diagnósticos de ICD 10 (ver anexo) y descrito en la sección de metodología.

METODOLOGÍA

Se realizará un ensayo aleatorizado para evaluar el impacto de la capacitación PRONTO^2. El diseño para la evaluación contempla la existencia de dos brazos. Un brazo con un grupo de hospitales que serán intervenidos con el programa PRONTO^2, denominado grupo tratamiento y un segundo brazo con un grupo de hospitales que no serán intervenidos en la primera fase de la implementación del programa y que se denominará grupo control. Se aleatorizará la selección de los hospitales a los grupos control y tratamiento previo un proceso de pareo de los hospitales más parecidos de acuerdo a

criterios específicos. Se contempla el análisis de indicadores de impacto y de proceso que serán colectados en los años basal, seguimiento intermedio (a los tres meses) y seguimiento 1 (a los seis meses de haber realizado la intervención).

Etapa 1: Selección de la muestra

La primer etapa del diseño consiste en seleccionar una muestra aleatoria simple (M) de n hospitales de la lista de hospitales resolutivos del Centro Nacional de Equidad de Género y Salud Reproductiva de la Secretaría de Salud de los estados de Guerrero, Chiapas y Estado de México donde el número de partos y cesáreas atendidas fue menor a 3,000 en el año 2009 (partos entre 500 y 3000 universo). La decisión de restringir las entidades donde evaluar a PRONTO^2 se debe a las facilidades ofrecidas por las autoridades de salud de esos estados para la evaluación de PRONTO^2; adicionalmente, la elección de hospitales con menos de 3,000 partos y cesáreas se fundamentó en la idea de que la capacitación ofrecida por PRONTO^2 sea la más adecuada y efectiva, ya que la capacitación del personal en hospitales grandes podría limitar el alcance de la intervención.

El número de hospitales en los estados de Chiapas, Guerrero y Estado de México con más de 500 partos y menos de 3,000 partos/cesáreas es 24; por tanto, el número máximo de pares a formar es de 12, esto es un tamaño de muestra aproximado de 12x700 = 8400.

Etapa 2: Pareo de Hospitales

La segunda etapa del diseño consiste en el pareo de hospitales, a cada hospital A en la muestra M se le asocia un hospital B que sea cercano (similar) con base en las variables listadas en la Tabla 1 que denotan tres aspectos: el tamaño del hospital medida a través del número de partos atendidos; el número de complicaciones atendidas por tipo y la capacidad de atención a través del número de personal médico e infraestructura disponible. De esta forma se forman n pares de hospitales con base en éstas características. El criterio usado para medir la cercanía entre dos hospitales A y B fue la distancia de Mahalanobis,

$$D = (X(A) - X(B))^T \Sigma^{-1} (X(A) - X(B))$$

Donde X() es el vector con los valores de las variables listadas en la Tabla 1 y Σ es la matriz de varianza de X(). Después de construir los n pares (A,B), a cada par se le asigna de modo aleatorio dos etiquetas, por ejemplo (A "grupo intervención" , B "grupo control"), etiquetas que indican si los hospitales conforman el grupo intervención que son aquellos que recibirán el programa PRONTO[2] y el grupo intervención que son los hospitales que no recibirán el programa.

Tabla 1. Variables que conforman el vector X

Variable	Definición
C_partos	Número de partos y cesáreas
Perinatal	Razón entre muertes perinatales y [partos+cesáreas]
Hemorragia	Razón entre casos de hemorragia obstétrica y [partos+cesáreas]
Hipertensiva	Razón entre casos de enfermedad hipertensiva y [partos+cesáreas]
Eclampsia	Razón entre casos de eclampsia y [partos+cesáreas]
Sepsis	Razón entre casos de sepsis y [partos+cesáreas]
Complicación	Razón entre [Perinatal + Hemorragia + Hipertensiva + Eclampsia+ Sepsis] y [partos+ cesáreas]
Enfermeras	Razón entre número de enfermeras y [partos+cesáreas]
Médicos	Razón entre número de médicos no especialista [médicos generales + pediatras] y [partos+cesáreas]
Médicos_E	Razón entre número de médicos especialistas [Gineco-obstetra + Cirujano + Anestesiólogo] y [partos+cesáreas]
Quirofanos	Razón entre número quirófanos y [partos+cesáreas]

Etapa 3: Colección de datos para la evaluación

Se revisarán y modificarán los instrumentos para la recolección de la información. Se colectará la información en todos los hospitales en dos momentos: en t=0 para tener una medida basal y en t=1 para medir los cambios observados (primer seguimiento). Como resultado del diseño propuesto, se espera colectar el conjunto de datos siguiente donde Y() es una variable de interés (ver Tabla 2). Levantamiento de proceso a los 6 meses, y de resultado a los 12 meses,

Tabla 2. Conjunto de datos a colectar, Y es la variable de interés

Hospitales	Basal (t=0)	Evaluación (t=1)
Intervención	$Y_I(0)$	$Y_I(1)$
Control	$Y_D(0)$	$Y_D(1)$

La información que se recolectará será:

1. Encuesta sobre el personal del hospital y recursos (visita hospitalaria y bases de datos del CNEGySR)
    a. Número de médicos y enfermeras
    b. Servicio y horario de laboratorio
    c. Capacidad del quirófano
    d. Medicamentos
    e. Equipo (ultrasonido, AMEU, Doppler) y localización y acceso del mismo
    f. Número de médicos generales
    g. Número de gineco-obstetras
    h. Número de pediatras
    i. Número de anestesiólogos
    j. Número de enfermeras

2. Recolección de indicadores epidemiológicos
    a. Número de nacimientos
    b. Número de cesáreas / número de partos vaginales
    c. Número de abortos
    d. Número de legrados
    e. Casos de eclampsia
    f. Casos de hemorragia obstétrica

g. Casos de preeclampsia
h. Muertes maternas
i. Muertes perinatales
j. Número de casos de histerectomía obstétrica
k. Casos admitidos en la unidad de cuidados intensivos
l. Casos admitidos en cuidados intensivos neonatales
m. Casos de complicaciones maternas referidos
n. Casos de complicaciones neonatales referidos
o. Referencias por hemorragia obstétrica u otra complicación materna severa
p. Número de transfusiones
q. Incidencia de hemorragias obstétricas
r. Letalidad por hemorragia obstétrica (#muertes debidas a hemorragias obstétricas/#total de hemorragias obstétricas)
s. Letalidad por preeclampsia/eclampsia (#muertes debidas a preeclampsia/eclampsia / #total de preeclampsia/eclampsia)
t. Relación de casos de preeclampsia derivados en eclampsia
u. # de casos de histerectomía obstétrica / número total de partos
v. Admisión de casos obstétricos a la unidad de cuidados intensivos
w. Admisión de neonatos a la unidad de cuidados intensivos
x. Uso de incubadora/# total de nacimientos
y. # de referencias por complicaciones severas
z. # de casos que necesitaron reanimación neonatal / número total de partos

3. Cuestionarios personales.
   a. Trabajo en equipo
   b. Auto-confianza
   c. Auto-eficacia
   d. Evaluación de conocimientos (hemorragia, reanimación neonatal, Preeclampsia/Eclampsia, distocia de hombro y presentación pélvica)

4. Observación de partos. Para recolectar información sobre el trabajo en equipo y obtener información sobre las siguientes prácticas:
    a. Manejo activo de la tercera etapa del parto (MATEP)
    b. Pinzamiento tardío de cordón
    c. Contacto materno/infantil temprano
    d. Episiotomía de rutina
    e. Limpieza uterina
    f. Presión fundal (Kresteller)

5. Otra información clave.
    a. Distancia al hospital de referencia
    b. Número de unidades de referencia
    c. % de pacientes derechohabientes
    d. % de personas indígenas

La recolección de la información de los 12 pares de hospitales, se realizará en 3 fases, en la primera se cubrirán 4 pares de hospitales, en la segunda 6 pares y en la tercera 2 pares.

Etapa 4. Implementación de la intervención

Se comenzará con la implementación en los hospitales de intervención en los estados selecionados, una vez recolectada la información de la línea basal.

Al igual que la recolección de la información, la implementación se realizará también en tres fases, en la primera se cubrirán 4 hospitales de intervención, en la segunda 6 hospitales de intervención y en la tercera 2 hospitales.

La implementación se realizará en dos momentos:

a. PRONTO[2] I (Primera visita. Duración de 2 a 4 días). Tiempo 0, después de haber recolectado información de línea basal.
    i. Trabajo en equipo
    ii. Comunicación
    iii. Hemorragia Obstétrica (aborto, atonía, placenta previa, laceración cervical, hemorragia posparto)

iv. Reanimación neonatal (con meconio y sin meconio)

b. PRONTO^2 II (Segunda visita. Duración 1 a 2 días) Tiempo de 2 a 3 meses después de la primera visita.

i. Reforzar trabajo en equipo

ii. Reforzar comunicación

iii. Revisión de hemorragia obstétrica y reanimación neonatal

iv. Introducción del tema de Preeclampsia/eclampsia

v. Introducción del tema de Distocia de hombros/Presentación pélvica

Descripción de la intervención

Las metas de la intervención PRONTO^2 son:

1. Mejorar el trabajo en equipo entre los proveedores encargados de atender los partos y las emergencias obstétricas y neonatales.

2. Mejorar la comunicación entre los miembros del equipo y reconocer e identificar habilidades y conocimientos de cada miembro del equipo.

3. Brindar elementos basados en evidencia científica para realizar la atención de las emergencias obstétricas de manera óptima y oportuna.

PRONTO^2 es una intervención que se desarrolla en dos módulos. El módulo I tiene una duración de dos o tres días y se realiza dentro del hospital por medio de simulaciones con alta fidelidad y utilizando baja tecnología, para alcanzar las metas de la intervención. El curriculum utiliza elementos del programa Team STEPPS para fortalecer el trabajo en equipo, liderazgo y el uso de técnicas efectivas de comunicación. Las actividades y escenarios tienen objetivos claros con conceptos de medicina basada en evidencia.

Los componentes del módulo I incluyen:

1. Curso virtual. Este es un curso por medio de diapositivas que estará disponible como curso virtual en el sitio de internet del INSP y será de acceso público. Este curso incluirá información didáctica crítica basada en evidencia, y algoritmos de manejo de hemorragia obstétrica y reanimación neonatal.

2. Dinámicas. Para fortalecer el trabajo en equipo, liderazgo y técnicas de comunicación.

3. Sesión de habilidades. Se revisan técnicas de reanimación neonatal con un maniquí, se practica sutura B Lynch y otras suturas para taponamiento uterino; así como el uso de sonda Foley, y el cálculo de pérdida de sangre.

4. Sesión de planes estratégicos para el hospital. Los participantes definen acciones factibles y específicas para llevar a cabo en el hospital en un determinado tiempo, para mejora el manejo de hemorragias obstétricas y reanimación neonatal.

5. Simulaciones. Se realizan 6 simulaciones que ejemplifican las principales causas de hemorragia obstétrica (atonía inmediata posparto, atonía severa posparto, placenta previa, aborto incompleto, laceración cervical), sufrimiento fetal y aspiración de meconio.

6. Pre y post evaluación del hospital

7. Pre y post evaluación de los participantes

El módulo II, corresponde al seguimiento de la capacitación, utiliza la misma metodología de aprendizaje que el Módulo I para reforzar el trabajo en equipo, comunicación y manejo de la emergencia, e incorpora temas nuevos que son preeclapsia/eclampsia, distocia de hombros y presentación pélvica.

Los componentes del módulo II son:

1. Curso virtual sobre preeclampsia/eclampsia, distocia de hombros y presentación pélvica
2. Dinámicas para reforzar el trabajo en equipo, liderazgo y comunicación
3. Sesión de Habilidades (maniobras de manejo de distocia de hombros y de presentación pélvica, repaso de algoritmos de manejo de pre-eclampsia/eclampsia)
4. Sesión de plan estratégico, para dar seguimientos a las metas que se establecen en el Módulo I, y detectar barreras para realizar los cambios en el hospital
5. Simulaciones que tocan los temas de distocia de hombros, presentación pélvica, hemorragia, eclampsia y reanimacion neonatal

6. Pre y Post evaluaciones de los participantes

Se espera que la intervención PRONTO[2] tenga un impacto directo en cada una de las variables mencionadas en la sección de preguntas de investigación. El presupuesto sería muy elevado si se capacitara al 100% del personal de cada uno de los hospitales, por lo que se estima que si se capacita al 30% del personal que atiende los casos obstétricos en cada hospital (médicos generales, especialistas, enfermeras) se tendrá el poder suficiente para detectar diferencias en los indicadores de interés.

Etapa 5: Análisis

Dado que la asignación de los hospitales a los grupos de intervención diferida e inmediata se realizará de manera aleatoria, los valores esperados de YD(0) y YI(0) son los mismos; por tanto, la diferencia en diferencias (Δ) es utilizada como una medida del efecto del programa PRONTO porque Δ compara el cambio en el grupo de intervención inmediata con el cambio en el grupo control.

$$\Delta = E\{ YD(1) \} - E\{ YD(0) \} - (E\{ YI(1) \} - E\{ YI(0) \}),$$

La diferencia de diferencias Δ es una suma de valores esperados. La diferencia de diferencias debe ser estimada tomando en cuenta el efecto de diseño, que es un factor que sirve para ajustar la varianza cuando se hace una selección de hospitales y no solamente una selección de partos/cesáreas. Adicionalmente se considera el coeficiente de correlación intra-conglomerado, que se refiere al número de elementos promedio dentro de cada conglomerado (es decir los partos dentro de un hospital). En este caso se está considerando el número efectivo de partos/cesáreas por conglomerado después de descontar el efecto del muestreo por conglomerados (cada conglomerado es un hospital).

La tabla 1 muestra la lista de resultados (Y) que se evalúan, los valores de PD1 y el coeficiente de correlación intra-conglomerados (□). La Tabla 1 sugiere que es suficiente con considerar los valores de 1%, 5% y 10% para el efecto diseño (PD1). Una vez obtenidas estimaciones para las tasas basales del efecto diseño (PD1) y el coeficiente de correlación intra-conglomerado □, es posible calcular la potencia que se espera obtener de este estudio cuando se colecta información por un período de seis meses y se prueba la hipótesis nula H: Δ=0 contra la hipótesis alternativa H: Δ<0.

La Tabla 3 muestran que con un tamaño de muestra de 8,400 (12 pares de hospitales), el único efecto detectable con una potencia cercana al 60% sería una reducción del 75% en PD,1 cuando el PD,1es cercana a 0.10.

Tabla 3. Poder de la prueba para detectar un decremento del 75%

$P_{D,1}$	r	Deff	N 3500	4200	4900	5600	6300	7000	7700	8400
1%	0.02	15.9	0.11	0.12	0.13	0.14	0.16	0.17	0.18	0.19
5%	0.05	38.4	0.18	0.20	0.22	0.25	0.27	0.29	0.31	0.33
10%	0.05	38.4	0.30	0.35	0.39	0.44	0.48	0.51	0.55	0.58

Nota. El valor del Deff se obtuvo al suponer solo se colectará información por medio año. El número promedio de partos y cesáreas esperado por semestre es de 1454/2. Nivel de la prueba 5%.

Consideraciones éticas

Antes de comenzar la capacitación se le presentará a cada uno de los participantes el consentimiento informado, el cuál contiene la descripción del estudio, la información relacionada con su participación, así como el derecho que tienen de abandonar el estudio en el momento que lo decidan. Se les proporcionará a cada participante una tarjeta con la información de contacto de la investigadora principal y de la presidenta de la comisión de ética del INSP.

Los cuestionarios de la línea basal, así como los de seguimiento tendrán un número de folio en la parte superior derecha para poder identificarlos, y se guardarán en la oficina de la investigadora principal. Asimismo, las video grabaciones de las simulaciones que se realicen, serán identificadas con un número de folio y serán resguardadas bajo llave en la oficina de la investigadora principal.

Solamente el personal involucrado en el estudio tendrá acceso a los cuestionarios y a las video-grabaciones.

CRONOGRAMA

Etapas	2010						2011						2012						2013		
	Bimestres						Bimestres						Bimestres						Bimestres		
	1	2	3	4	5	6	1	2	3	4	5	6	1	2	3	4	5	6	1	2	3
**Etapa 1.** Selección de la muestra	x																				
**Etapa 2.** Pareo de Hospitales		x																			
**Etapa 3.** Colección de datos. Línea basal																					
**-Fase 1:** 4 pares hospitales			x	x																	
**-Fase 2:** 6 pares hospitales							x	x													
**-Fase 3:** 2 pares hospitales													x								
**Etapa 3.** Colección de datos. Seguimiento 3, 6 y 12 meses																					
**-Fase 1:** 4 pares hospitales				x	x		x		x	x			x								
**-Fase 2:** 6 pares hospitales										x	x		x	x							
**-Fase 3:** 2 pares hospitales														x		x		x			
**Etapa 4.** Implementación intervención. Módulo I y Módulo II																					
**-Fase 1:** 4 hospitales			x	x		x	x														
**-Fase 2:** 6 hospitales							x	x	x	x											
**-Fase 3:** 2 hospitales														x		x		x			
**Etapa 5.** Análisis						x	x				x	x				x	x	x	x	x	x

Referencias

1. Gobierno de los Estados Unidos Mexicanos. Presidencia de la República. Programa Nacional de Salud 2007-2012. Capítulo IV, Seguimiento de metas, medición de resultados y rendición de cuentas. Primera edición 2007. p. 131-151 (Disponible en: http://portal.salud.gob.mx/sites/salud/descargas/pdf/pnscap4.pdf).

2. Mortalidad Materna en México. Monitoreo Ciudadano de la Política Pública Federal para Reducir la Morbimortalidad Materna en México. Capítulo Distrito Federal. Marzo 2005-mayo2006. (Disponible en: http://www.mortalidadmaterna.com.mx/docs/Monitoreo.pdf).

3. María del Carmen Elú, "Iniciativa para una Maternidad sin riesgos en México", en Graciela Freyermuth y María Manca, Luna golpeada. Morir durante la maternidad: investigaciones, acciones y atención médica en Chiapas y otras experiencias en torno a la mortalidad materna, Comité promotor por una maternidad sin riesgos en México, México, 2000, p. 14.

4. Langer Ana. "La Mortalidad materna en México: la contribución del aborto inducido", en Ortiz A. (ed.), Razones y pasiones en torno al aborto, México, Edamex/Population Council, 1994, pp.149-153.

5. Organización de las Naciones Unidas. Objetivos de Desarrollo del Milenio. (Disponible en: http://www.objetivosdelmilenio.org.mx/).

6. Bergonzoli G. Mortalidad materna y neonatal relación ODM-OPS. (Disponible en: www.lachealthsys.org/.../ _ODMs-OPS-Gustavo%20Bergonzoli.pps).

7. Lozano AR, Suárez VA. Mortalidad neonatal. Bol Med Hosp Infant Mex 2004;61:275-9)

8. American Academy of Pediatrics y American Heart Association. (2006). Reanimacion Neonatal. (5th ed).

9. Sistema Nacional de Información Estadística y Geográfica (INEGI) y Dirección de Informática y Geografía Médica. Núcleo de Acopio y Análisis de Información en Salud. INSP con datos obtenidos de la Secretaría de Salud. Se analizó a nivel estatal, con datos de la Encuesta Nacional de Salud 2000 y la Encuesta Nacional de Salud y Nutrición 2006.

10. Instituto Nacional de Estadística, Geografía e Informática (INEGI). Conteo 1995 y 2005, XII Censo General de Población y Vivienda 2000. Procesada por Instituto Nacional de Salud Pública INSP

11. Steven G. Gabbe, MD, Jennifer R. Niebyl, MD, Joe Leigh Simpson, MD, Henry Galan, MD, Laura Goetzl, MD, MPH, Eric R.M. Jauniaux, MD, PhD, and Mark Landon, MD Obstetrics: Normal and Problem Pregnancies, 5th Edition. Elsevier publishers. ISBN: 9780443069307

12. Deborah Maine Detours and shortcuts on the road to maternal mortality reduction Viewpoint. Lancet 2007; 370: 1380–82

13. Otchere SA, Kayo A. The challenges of improving emergency obstetric care in two rural districts in MaliInt J Gynaecol Obstet. 2007 Nov;99(2):173-82. Epub 2007 Sep 27

14. M. Kayongo, J. Butera, D. Mboninyibuka, B. Nyiransabimana, A. Ntezimana, V. Mukangamuje. Improving availability of EmOC services in Rwanda — CARE's experiences and lessons learned at Kabgayi Referral Hospital Int J Gynaecol Obstet. 2006 Mar;92(3):291-8.

15. Kayongo M, Esquiche E, Luna MR, Frias G, Vega-Centeno L, Bailey P Strengthening emergency obstetric care in Ayacucho, Peru Int J Gynaecol Obstet. 2006 Mar;92(3):299-307.

16. Department of Health. Working for patients. London: HMSO, 1989.

17. Kongnyuy EJ, Leigh B, van den Broek N Effect of audit and feedback on the availability, utilisation and quality of emergency obstetric care in three districts in Malawi Women Birth. 2008 Dec;21(4):149-55

18. Muffler N, Trabelssi Mel H, De Brouwere V. Scaling up clinical audits of obstetric cases in Morocco Trop Med Int Health. 2007 Oct;12(10):1248-57.

19. Cohen S.R., Cragin, L., Walker, D.M. datos aún no publicados

20. WHO/ICM/FIGO, 2005

21. Davis D, Thomson O'Brien MA, Freemantle, Wolf FM, PhD; Mazmanian P, Taylor-Vaisey. A Impact of Formal Continuing Medical Education Do Conferences, Workshops, Rounds, and Other Traditional Continuing Education Activities Change Physician Behavior or Health Care Outcomes? JAMA. 1999;282 (9):867-874.

22. Halamek LP,Kaegi DM, Gaba DM, Sowb YA, Smith BC, Smith BE, Howard SK Time for a New Paradigm in Pediatric Medical Education: Teaching Neonatal Resuscitation in a Simulated Delivery Room Environment PEDIATRICS Vol. 106 No. 4 October 2000, p. e45 ELECTRONIC ARTICLE

23. W R Hamman The complexity of team training: what we have learned from aviation and its applications to medicine Quality and Safety in Health Care 2004;13(Supplement 1):i72-i79; doi:10.1136/qshc.2004.009910 Copyright © 2004 by the BMJ Publishing Group Ltd.

24. SSM Health Care's Foundation of Safety and Care. STEPPS: Producing Effective Medical Teams to Achieve Optimal Patient Outcomes AHRQ Annual Conference Sept. 10, 2008

25. JamtvedtG, Young JM, KristoffersenDT, Thompson O'Brien MA, OxmanAD. Audit and feedback: effects on professional practice and health care outcomes. TheCochrane Database SystRev2003; 3: CD000259. DOI: 10.1002/14651858.CD000259.
