# Supplementary material for: Can a simulation-based training program impact the use of evidence based routine practices at birth? Results of a hospital-based cluster randomized trial in Mexico
Source: PLoS One. 2017 Mar 20;12(3):e0172623. doi: 10.1371/journal.pone.0172623 (PMC5358738; doi:10.1371/journal.pone.0172623)
Supplement: S2 Protocol — (PDF) [file pone.0172623.s003.pdf]

RESEARCH PROJECT PROTOCOL

PRONTO "Program of Obstetric and Neonatal Rescue, Optimal & Timely Treatment. A Cluster-Randomized Trial for an Impact Evaluation"

Principal Investigator:

Dilys Walker, MD

Associate Professor University of Washington Depts. of Ob Gyn and Global Health

Team:

Dra. Sandra Gabriela Sosa Rubí, National Institute of Public Health

Dr. Martín Romero, National Institute of Public Health

CNM Susanna Cohen, University of Utah School of Nursing

CNM, MPH Jennifer Fahey, University of Maryland School of Medicine

Mtra. Dolores González Hernández, National Institute of Public Health

ABSTRACT

Rates and causes of maternal mortality in Mexico have dropped only slightly; thus, reaching the internationally established Millennium Development Goals (MDG) is still a distant goal for this country. A fundamental part of reducing maternal and infant mortality is ensuring an adequate and timely response to obstetric emergencies. PRONTO^2: Obstetric and Neonatal Emergency Training Program is an innovative training strategy based on simulations designed to train hospital personnel to respond to obstetric emergencies. The objective of this study is to implement PRONTO^2 in randomly selected hospitals to measure the effectiveness of the intervention in influencing key behaviors in hospital practices, as well as measuring maternal and neonatal outcomes in intervention versus control hospitals.

INTRODUCTION

Rates and causes of maternal mortality in Mexico have dropped only slightly; thus, reaching the internationally established Millennium Development Goals (MDG) is still a distant goal. PRONTO^2: Obstetric and Neonatal Emergency Training Program is an innovative training strategy based on simulations designed to train hospital personnel to respond to obstetric emergencies.

Many evaluations on maternal mortality have indicated that the quality of obstetric services as well as providing timely and appropriate care of obstetric emergencies is a key to reduce rates of maternal morbidity and mortality in the hospital environment. Traditional training models as didactic sessions or the introduction of guidelines and protocols have not shown the expected results in the indicators of interest. PRONTO^2 uses a unique methodology of its kind in Mexico, with a simulation model (high fidelity and low technology) to train multidisciplinary teams attending obstetric emergencies in hospitals in order to respond to emergencies in an effective and successful manner, integrally incorporating elements of knowledge, practices, availability of inputs and hospital infrastructure and health systems.

In 2009 the team of PRONTO^2 (Spanish acronym for: *Programa de Rescate Obstétrico y Neonatal: el Tratamiento Óptimo y Oportuno;* ^{2} for the last 2 "O") developed the curriculum, implementation manuals and conducted a pilot study in two hospitals in the state of Mexico and in two hospitals in the state of Chiapas, which were selected by Ministries of Health in each of the states. The pilot study consisted in the PRONTO^2 curriculum implementation for hospital staff, covering 15 hours of training. At the end of the implementation, a report was handed over to each hospital, pointing specific and feasible goals established by the participants, to improve emergency obstetric care in hospitals. The preliminary results of this pilot and follow-up at 3 months of the implementation, not only has shown the feasibility of carrying out the PRONTO^2 intervention in situ, but was recorded the acceptability by the Secretaries of Health, hospital directors, as well as by participants from both the methodology of learning through simulations, and changes observed in participants following the implementation of PRONTO^2.

Therefore, the next step is to conduct a randomized and controlled implementation to evaluate the program in a rigorous manner, to assess the impact of PRONTO[2] in practices of obstetric and neonatal emergency outcomes in short and long term, as well as teamwork and communication among members of the medical team and the patient.

BACKGROUND

Maternal mortality is a major public health problem in our country. While in recent years it has documented a decline in maternal mortality, going from 89 deaths per 100,000 live births in 1990 to 63.4 in 2005 according to official statistics; there are still regions of the country where the rates are still high, especially in states such as Guerrero, Oaxaca and Chiapas.[1,2]

Maternal mortality is a good indicator of socioeconomically conditions of women. [3]

Although the frequency of maternal deaths in absolute numbers is low compared with other public health problems, behind the death of a woman there are serious consequences such as family disintegration, high infant mortality and morbidity, dropouts, malnutrition, orphans and premature entry of children into the labor market.[4]

Given this problem, the health sector has implemented several actions at various levels to reduce maternal mortality. Reducing maternal mortality is one of the Millennium Development Goals (MDG) signed by our country. Specifically, it is proposed to reduce goal four, between 1990 and 2015, maternal mortality by three quarters. [5]

For ten years to complete the time set in the MDG progress was less than 45%.[1] Maternal mortality is the 4th leading cause of death in women of reproductive age in Mexico and mainly occurs from causes related to obstetric emergencies: 25% hypertensive disease of pregnancy and 24% for bleeding during pregnancy and childbirth. [1]

Also, the fourth Millennium Development Goal proposes a reduction in infant mortality by two thirds, between 1990 and 2015, Mexico currently is five percentage points below the target.[6] In Mexico, more than half of the deaths in children under 5 years are perinatal and occur within the first 24 hours of life. Conducting hospital interventions focused on neonatal resuscitation in the first minute of life are effective in reducing neonatal

morbidity and mortality.[7,8] According to data from 2004, 12 states account for 75% of all maternal deaths: Chiapas, Guerrero, Oaxaca, Puebla, Durango, Veracruz, Hidalgo, the state of México, San Luis Potosí, Baja California, Mexico City and Querétaro.[2]

Unlike the situation in previous years (at least until the 90s), a high proportion of maternal deaths occur in hospital units.[9]

A recent analysis of data from National Institute for Statistics and Geography (INEGI 2005) found that in almost 3/4 of the country's municipalities the proportion of births attended by medical units is over 70%.[10]

Effective strategies to battle maternal mortality and morbidity.

There have been developed and evaluated different strategies to influence the reduction of maternal and perinatal mortality. Since it is a complex issue, there is no universal strategy to combat or reduce rates of maternal and perinatal mortality.

Some of the relevant strategies are mentioned below:

1. Improve the quality of medical care for pregnant women, especially when facing an obstetric emergency. An essential element for reducing maternal mortality is to provide quality health services during pregnancy and birth.

A good proportion of maternal deaths occur at birth, diagnosed with obstetric hemorrhage and preeclampsia / eclampsia, so that providing timely and appropriate care of obstetric emergencies is the key to lower rates of maternal mortality in the hospital environment. Often these complications do not appear until labor or birth. Overall about 10% of deliveries will health any complication for one reason or another.[11] Also, there are estimations that show that care during childbirth and in the first hours of life of newborn are critical for their survival.

Numerous maternal mortality assessments have indicated that the quality of obstetric care is an important issue that must be addressed. The systematic improvement of these services in developing countries such as Malaysia and Sri Lanka, have managed to bring down maternal mortality rates by up to 50% over a period of 7 to 10 years.[12]

2. Strengthen infrastructure of medical units. It is possible to provide quality obstetric services in communities but it is necessary to strengthen the needed infrastructure.[13]
There are examples of regional hospitals in Rwanda, Africa where the renovation of hospitals with equipment and staff training, resulted in a qualified hospital to treat obstetric emergencies with fewer complications, maternal and child deaths, without altering their numbers of Caesarean sections.[14]
In Latin America there are examples such as Peru, where improved facilities and services for emergency obstetric care yielded positive results in reducing maternal mortality[15]

3. Periodic audits with feedback to measure quality of care. To evaluate the quality of care there have been implemented several years ago audits, which are defined as the systematic critical analysis of the quality of care and includes diagnostic procedures, treatment, use of resources and the quality of life of patients.[16]
It is considered that the audits with feedback services can help improve resource utilization and quality of emergency obstetric care, especially in countries with limited resources.[17] To achieve the implementation of clinical audits, health services authorities should invest in training and monitoring of their medical services. [18]

4. Training with new techniques such as simulations. In previous studies from 2004 to 2007 by the research group led by Dr. Dilys Walker, they assessed the quality of care offered by different providers associated with obstetric care: general practitioners, obstetricians, nurses and technical professional midwives. In this context, a strategy was developed to train these providers in emergency obstetric care, using simulations in clinical practice based on scientific evidence and in deficiencies identified in previous assessments that these providers had in their obstetric practice. An evaluation of this training showed that the knowledge and self-efficacy increased significantly and remained until 3 months after training.[19]

Our experience to train health personnel linked to care delivery using simulations in 2009, and the implementation of PRONTO^2 in four hospitals, gives us the technical support and expertise to develop this new phase of research that includes the development of a research study called: **PRONTO^2 Program of Obstetric and Neonatal Rescue**

Training Emergency: A Cluster-Randomized Trial for an Impact Evaluation. The study will be directed to implement the intervention in a sample of randomly selected hospitals (described below) to train an interdisciplinary group of staff assigned to hospitals selected by the Ministry of health linked to emergency obstetric care in secondary and tertiary care hospitals.

PRONTO^2 will use a learning technique based on the use of simulations for handling obstetric emergencies in the hospital environment. This strategy seeks to improve the quality of care offered in the case of an obstetric emergency, for which information will be obtained in three stages of obstetrical and neonatal outcomes as well as process for conducting an impact assessment of the program PRONTO^2.

RATIONALE

Adequate and timely emergency obstetric care is fundamental for the reduction of maternal and infant mortality.[20] Since most maternal deaths in Mexico occur in the hospital environment and there is evidence that quality of care offered is an important element in the causes of death, it is critical to develop and evaluate innovative strategies to improve the quality of care in the case of obstetric emergencies.

In this sense, we propose a strategy based on the use of simulations where the emergencies occur, trying to improve the quality of care.

So far, traditional methods of learning and teaching resources, such as teaching resources, brochures, guides and manuals, have not shown to have a high effectiveness in changing behavior of providers in using evidence-based practices.[21]

The frequency of occurrence of obstetric emergencies is low, so that health staff has not repetitive clinical practice that allows integrate their experiences, knowledge and skills to practical field. The learning method through simulations offers the possibility to participate in the recreation of a real clinical case where through life experience between the medical team it is possible to identify how they should act, interact and satisfactorily resolve an obstetric emergency.Simulation is an educational system adapted from the space aviation industry to medicine and it aims to increase knowledge, improve skills, to

form leadership and ensure the efficient mobilization of resources and proper coordination of health care. [22]

The simulation is defined as a training that aims to immerse the student in a real situation (scenario) created within a physical space (simulator) which replicates the real environment with such fidelity that the student believes it is real. Part of the methodology of the simulation includes filming the entire simulation to ensure accurate reproduction of the team behavior in an emergency. From a playback on a monitor in front of all personnel involved, an immediate feedback is performed. As well this allows observing and evaluating the response of the team in training: if healthcare to women are given in a coordinated manner and leadership skills that are manifest spontaneously.

Evaluation of the implemented strategies in various environments and in several countries to improve health care, have identified factors that had not previously been taken into account. A frequent mistake on health services, which compromises the safety of the patient, is linked to latent failures in the structure and function of systems. The care of patients is a responsibility of an integrated team of health care providers, so the current challenge for training programs for health professionals, it is to increase the experience of working in multidisciplinary teams. The complexity of these team training requires multifunctional systems that go beyond organizational divisions, enable communication, accountability and maintenance of the team itself.[23]

Nowadays training teamwork is crucial, especially in the areas of health care. PRONTO[2] incorporates elements of a program known as Team STEPPS, which directs its attention to solve communication problems and fragmentation of work. Training and evaluation is focused on the operation of the team, to ensure the safety of the patient.[24]

Because rules for evaluating obstetric emergency services' improvement are still under study, the methodology of this study, proposes to determine along with the hospital under study, those problems of clinical practice that might need solution, determine the standards of care for that problem so that practices are monitored and improved.[25]

Obtaining indicators in the short and long term are based on practical considerations. The selection of indicators in the short and long term will be selected depending on the

problems to be solved. Some examples would be the availability of blood in cases of postpartum hemorrhage, the availability and use of oxytocin in the active management of the third stage of labor, the availability and use of magnesium sulfate in severe preeclampsia, lower rates of caesarean section or hysterectomy.

GENERAL OBJECTIVE

The main aim of this study is to implement the PRONTO^2 intervention in randomly selected hospitals, and measure the effectiveness of this intervention in key behaviors of hospital practice in emergency obstetric care (process indicators), as well as collect information on obstetric and neonatal indicators (outcomes) in the intervention and control hospitals to conduct an impact evaluation of the program PRONTO^2.

SPECIFIC OBJECTIVES

- Measure the effect of the program PRONTO^2 in process indicators.
- Measure the impact of the program PRONTO^2 in obstetric and neonatal result indicators

Research questions:

1. Does PRONTO^2 training improve teamwork of the hospital staff attending obstetric emergencies?
2. Does PRONTO^2 training improve communication among team members attending obstetric emergencies?
3. Does PRONTO^2 training improve communication among the team attending obstetric emergencies and the patient?
4. PRONTO^2 increases and maintains through time levels on the scale of self-efficacy?
5. With PRONTO^2 training are changes observed in the structure and resources of the hospital? For example:

a. The place where the equipment (ultrasound, Doppler, refrigerator) and medications (oxytocin, ergonovine) is stored, as well as people who have access to them.

b. Visible access to management of obstetric and neonatal emergencies protocols (neonatal resuscitation algorithms, active management of the third stage of labor (AMTSL), use of drugs for obstetric hemorrhage)

c. Access to other resources such as:

    i. Misoprostol
    ii. Carbetocin
    iii. Foley
    iv. Hemocel

6. With PRONTO[2] training changes in hospital routines and practices are observed?

    a. Implementing the active management of the third stage of labor (AMTSL)
    b. Avoid supine position during labor
    c. Implementing delayed cord clamp
    d. Preventive measure for the presence of meconium (immediate aspiration, delay stimulation)
    e. Skin to skin contact
    f. Manuel vacuum aspiration (MVA) for treatment of abortion in the first trimester
    g. Avoid uterine sweeping
    h. Avoid fundal pressure (Kristeller)
    i. Decrease episiotomies

7. With PRONTO[2] training are any changes observed in indicators of obstetric and neonatal outcomes and epidemiological indicators? Selected from ICD 10 diagnoses (see appendix) and described in the methodology section.

METHODOLOGY

A randomized trial to evaluate the impact of the PRONTO2 training will be performed. The evaluation design contemplates the existence of two arms. An arm with a group of hospitals that will be intervened with the PRONTO2 program, called treatment group and a second arm with a group of hospitals that will not be intervened in the first phase of the program implementation called control group.

The selection of hospitals to control and treatment groups is at random prior to the process of matching most similar hospitals according to specific criteria. It includes the analysis of impact and process indicators that will be collected at baseline, intermediate follow-up (three months) and tracking one (six months after the intervention is performed).

Stage 1: Sample selection

The first stage of the design is to select a simple random sample (M) of n hospitals, from the operative list of the National Center for Gender Equity and Reproductive Health of the Ministry of Health of the states of Guerrero, Chiapas and Mexico, where the number of births and cesarean sections was less than 3,000 in 2009 (500 to 3000 universe of births).

The decision to restrict the states where to evaluate PRONTO 2, was due to the facilities offered by the health authorities of these states to assess PRONTO2. Additionally, the selection of hospitals with fewer than 3,000 births and c- sections was based on the idea that the training offered by PRONTO2 is the most appropriate and effective, as the training in large hospitals may limit the scope of the intervention.

The number of hospitals in the states of Chiapas, Guerrero and Mexico, with more than 500 births and fewer than 3,000 births / cesarean sections is 24; therefore, the maximum number of pairs to be formed is 12, this is a sample size of approximately 12x700 = 8400.

Stage 2: Matching hospitals

The second stage of the design consists in matching the hospitals. Each hospital A in the sample M is associated with a hospital B that is close (similar) based on the variables listed in Table 1 denoting three aspects: hospital size measured by the number of births attended; the number of complications treated by type; and the capacity of care through the number of medical personnel and infrastructure available. In this way, n pairs of hospitals are formed based on these characteristics. The criteria used to measure the closeness between two hospitals A and B was the Mahalanobis distance,

$$D = (X(A) - X(B))^T \Sigma^{-1} (X(A) - X(B))$$

Where X ( ) is the vector with the values of the variables listed in Table 1 y Σ it is variance matrix of X ( ).

After building the n pairs (A, B), to each pair is randomly assigned two labels, for example (A "intervention group" B "control group"), labels indicating whether hospitals form the intervention group, who are those who receives the program PRONTO 2, and the control group, hospitals that do not receive the program.

Table 1. Variables conforming the X vector

Variable	Definition

C_partos	Number of births and c sections
Perinatal	Ratio of perinatal deaths and [cesarean sections + births]
Hemorragia	Ratio of cases of obstetric hemorrhage and [births + cesarean sections]
Hipertensiva	The ratio of cases of hypertensive disease and [c sections + births]
Eclampsia	The ratio of cases of eclampsia and [births + c sections]
Sepsis	The ratio of cases of sepsis and [births + c sections]
Complicación	Ratio between [Perinatal + hemorrhage + hypertensive + eclampsia + Sepsis] and [births + c sections]
Enfermeras	Ratio of nurses and [births + c sections]
Médicos	Ratio of number of non-specialist physicians [general physicians + pediatricians] y [births + c sections]
Médicos_E	Ratio of number of specialists [OB-GYN Surgeon + + Anesthesiologist] y [births + c sections]
Quirofanos	Ratio of operating rooms and [births + c sections]

Step 3: Data collection for evaluation

Instruments for data collection will be reviewed and modified. Data will be collected in all hospitals at two moments: t=0 to have a baseline measure and t=1 to measure the changes observed (first follow up). As a result of the proposed design, is expected to collect the next set of data where Y()is a variable of interest (see Table 2). Process recollection at 6 month and outcome recollection at 12 months.

Table 2. Set of data to collect, Y is the variable of interest

Hospitals	Baseline (t=0)	Evaluation (t=1)
Intervention	$Y_I(0)$	$Y_I(1)$
Control	$Y_D(0)$	$Y_D(1)$

The information collected will be:

1. Survey for the hospital staff and resources (Hospital visit and databases from CNEGySR)

    a. Number of doctors and nurses
    b. Service and laboratory hours
    c. Operating room capacity
    d. Medicines
    e. Equipment (ultrasound, MVA, Doppler) location and access
    f. Number of general physicians
    g. Number of obstetricians
    h. Number of pediatricians
    i. Number of anesthesiologists
    j. Number of nurses

2. Collection of epidemiological indicators
    a. Births
    b. Number of C-sections / number of vaginal
    c. Number of curettage
    d. Cases of eclampsia
    e. Cases of obstetric hemorrhage
    f. Cases of preeclampsia
    g. Maternal deaths
    h. Perinatal deaths
    i. Number of cases of obstetric hysterectomy
    j. Cases admitted to the intensive care unit
    k. Cases admitted to neonatal intensive care
    l. Referred cases of maternal complications
    m. Cases of referred neonatal complications
    n. References by obstetric hemorrhage or other severe maternal complication

o. Number of transfusions
p. Incidence of obstetric hemorrhage
q. Obstetric hemorrhage lethality (#deaths due to obstetric hemorrhage /# total of obstetric hemorrhage)
r. Preeclampsia/eclampsia lethality (#deaths due preeclampsia/eclampsia / #total of preeclampsia/eclampsia cases)
s. List of cases of preeclampsia derived from eclampsia
t. # cases of obstetric hysterectomy / total number of deliveries
u. Admission of obstetrical cases to the intensive care unit
v. Incubator usage /# total births
w. # referrals for severe complications
x. # of cases requiring neonatal resuscitation / Total number of births

3. Personal Surveys.
    a. Teamwork
    b. Self-confidence
    c. Self Efficacy
    d. Knowledge assessment (hemorrhage, neonatal resuscitation, preeclampsia / eclampsia, dystocia and breech presentation)
4. Observation of deliveries.. To collect information on teamwork and information on the following practices:
    a. Active management of the third stage of labor (AMTSL)
    b. Delayed cord clamping
    c. Early maternal/child contact
    d. Routine episiotomy
    e. uterine cleaning
    f. Fundal pressure (Kristeller)

5. Other key information.
    a. Distance to the referral hospital
    b. Number of reference units
    c. % of patients right holders

d. % indigenous people

Collecting information from 12 pairs of hospitals, will be held in 3 phases, in the first four pairs of hospitals will be covered, in the second 6 pairs and the third 2 pairs.

Stage 4. Implementation of the intervention

The implementation will start in the intervention hospitals of the selected states, once the baseline information is collected.

Just as the collection of information, implementation is also carried out in three phases, in the first one four intervention hospitals will be covered, 6 hospitals in the second intervention and two hospitals in the third one

The implementation will take place in two moments:

a. PRONTO2 I (First visit. Duration of 2 to 4 days). Time 0, after collecting baseline information.

i. Teamwork

ii. Comunication

iii. Obstetric Hemorrhage (abortion, atony, placenta praevia, cervical laceration, postpartum hemorrhage)

iv. Neonatal resuscitation (with and without meconium)

b. PRONTO2 II (Second visit. Duration 1 to 2 days) Time 2-3 months after the first visit.

i. Strengthening of teamwork

ii. Strengthening of communication

iii. Review of obstetric hemorrhage and neonatal resuscitation

iv. Introducing the topic of Pre-eclampsia / eclampsia

v. Introducing the topic of shoulder dystocia / pelvic Presentation

Description of the intervention

The goals of the PRONTO2 intervention are:

1. Improve teamwork among providers responsible for attending births and obstetric and neonatal emergencies.

2. Improve communication between team members and recognize and identify skills and knowledge of each one.

3. Provide evidence-based elements to attend obstetric emergencies in an optimal and timely manner.

PRONTO2 it is an intervention that is developed in two modules. Module 1 is delivered over 2-3 days, takes place in the hospital, and consists of high-fidelity, low-tech simulations. The curriculum employs elements of the Team STEPPS program to strengthen teamwork and leadership skills, and the use of effective communication techniques. The activities and simulations have clear objectives and use evidenced-based medical concepts. Module 2, the follow-up training, uses the same teaching methodology as Module 1 to reinforce teamwork, communication, and emergency response skills, and incorporates new topics such as preeclampsia /eclampsia, shoulder dystocia and pelvic presentation. Activities and scenarios have clear objectives with concepts of evidence-based medicine.

Module I components include:

1. Virtual Course. This course consists on slides that will be available as a virtual course on the website of the INSP and be publicly accessible. This course will include teaching critical evidence-based information and algorithms for the management of obstetric hemorrhage and neonatal resuscitation.

2. Group dynamics. To strengthen teamwork, leadership and communication skills.

3. Skills session. Neonatal resuscitation techniques are reviewed with a mannequin, B Lynch suture, and other sutures for uterine tamponade is practiced; and the use of Foley catheter, and calculation of blood loss.

4. Session for strategic plans for the hospital. Participants define feasible and specific actions to be carried out in the hospital at any given time, to improve the management of obstetric hemorrhage and neonatal resuscitation.

5. Simulations. Six simulations that illustrate the main causes of obstetric hemorrhage (immediate postpartum atony, severe postpartum atony, placenta praevia, incomplete abortion, and cervical laceration), fetal distress and meconium aspiration are performed.

6. Pre and post hospital evaluation

7. Pre and post participant's evaluation

Module II corresponds to the follow-up training, it uses the same learning methodology than Module I to strengthen teamwork, communication and emergency management and introduce new topics that are preeclampsia / eclampsia, dystocia and breech presentation.

Module II components are:

1. Virtual Course on preeclampsia / eclampsia, dystocia and breech presentation
2. Group dynamics to strengthen teamwork, leadership and communication
3. Skills Session (handling maneuvers dystocia and breech presentation, review of algorithms for the management of pre-eclampsia / eclampsia)
4. Strategic plan session, to give follow-up to the goals set out in Module I, and identify barriers to making changes in hospital
5. Simulations on the topics of shoulder dystocia, breech presentation, hemorrhage, eclampsia and neonatal resuscitation

6. Pre and post participant's evaluation

PRONTO2 intervention is expected to have a direct impact on each of the variables mentioned in the research questions section. To train 100% of the staff of each hospital would be very expensive, so it is estimated that if 30% of trained personnel attending obstetric cases in each hospital (General practitioners, specialists, nurses) we will have enough power to detect differences in the indicators of interest.

Step 5: Analysis

Since the allocation of hospitals to groups of deferred and immediate intervention will be carried out at random, the expected values for YD (0) and YI (0) are the same. Therefore, the difference in differences (Δ) is used as a measure of the effect of the PRONTO2 program, since (Δ) compares the change in the immediate intervention group with the change in the control group.

$$\Delta = E\{ YD(1) \} - E\{ YD(0) \} - (E\{ YI(1) \} - E\{ YI(0) \}),$$

The difference of differences Δ is a sum of expected values. The difference of differences should be estimated considering the design effect, which it is a factor used to adjust the variance when a selection of hospitals and not just a selection of delivery / c-sections is done.

Additionally it is considering the intra-cluster correlation coefficient, which refers to the average number of elements within each cluster (i.e. births in a hospital). In this case, it is considering the actual number of births / c-sections per cluster after discounting the effect of cluster sampling (each cluster is a hospital).

Table 1 shows the results list (Y) evaluated PD1 values and the intra-cluster correlation coefficient (ρ). Table 1 suggests that it is sufficient to consider the values of 1%, 5% and 10% for the design effect (PD1). After obtaining baseline rates estimates for the design effect (PD1) and the intra-cluster correlation coefficient ρ, it is possible to calculate the power expected from this study when information is collected for a period of six months and test the null hypothesis H: $\Delta=0$ against the alternative hypothesis H: $\Delta<0$.

Table 3 shows that with a sample size of 8,400 (12 hospitals pairs), the only detectable effect with a nearby power 60% would be a 75% reduction in PD 1 when the PD, near 1 is 0.10.

Table 3. Power of the test to detect a decrease of 75%

			N							
$P_{D,1}$	r	Deff	3500	4200	4900	5600	6300	7000	7700	8400
1%	0.02	15.9	0.11	0.12	0.13	0.14	0.16	0.17	0.18	0.19
5%	0.05	38.4	0.18	0.20	0.22	0.25	0.27	0.29	0.31	0.33
10%	0.05	38.4	0.30	0.35	0.39	0.44	0.48	0.51	0.55	0.58

Note. The value of Deff was obtained by assuming only collect information by year. The average number of births and c sections expected per semester is 1454/2. Level test 5%.

Ethical considerations

Before starting the training, informed consent is presented to each of the participants, which contains the description of the study, information relating to their participation, and the right they have to leave the study at the time they decide to do so.

Each participant will receive a card with the contact information of the principal investigator and the president of the Ethics Commission at the INSP.

The baseline and follow up questionnaires will have a folio number in the upper right corner for identification, and stored in the office of the principal investigator. Similarly, video recordings of the simulations carried out will be identified with a folio number and will be guarded locked in the office of the principal investigator.

Only staff involved in the study will have access to the questionnaires and the video-recordings.

SCHEDULE

Stages	2010						2011						2012						2013		
	Bimesters						Bimesters						Bimesters						Bimesters		
	**1**	**2**	**3**	**4**	**5**	**6**	**1**	**2**	**3**	**4**	**5**	**6**	**1**	**2**	**3**	**4**	**5**	**6**	**1**	**2**	**3**
**Stage 1.** Selection of the sample	x																				
**Stage 2.** Matching Hospitals		x																			
**Stage 3.** Data collection. baseline																					
**- Phase 1:** 4 hospitals pairs			x	x																	
**- Phase 2:** 6 hospitals pairs							x	x													
**- Phase 3:** 2 hospitals pairs													x								
**Stage 3.** Data collection. Follow up 3, 6 and 12 months																					

**-Phase 1:** 4 hospitals pairs				x	x		x		x	x			x								
**-Phase 2**: 6 hospitals pairs										x	x		x	x							
**- Phase 3**: 2 hospitals pairs														x		x		x			
**Stage 4.** Implementation intervention. Module I and Module II																					
**-Phase 1**: 4 hospitals			x	x		x	x														
**- Phase 2:** 6 hospitals							x	x	x	x											
**- Phase 3:** 2 hospitals														x		x		x			
**Stage 5.** Analysis						x	x				x	x				x	x	x	x	x	x

References

1. Gobierno de los Estados Unidos Mexicanos. Presidencia de la República. Programa Nacional de Salud 2007-2012. Capítulo IV, Seguimiento de metas, medición de resultados y rendición de cuentas. Primera edición 2007. p. 131-151 (Disponible en: http://portal.salud.gob.mx/sites/salud/descargas/pdf/pnscap4.pdf).

2. Mortalidad Materna en México. Monitoreo Ciudadano de la Política Pública Federal para Reducir la Morbimortalidad Materna en México. Capítulo Distrito Federal. Marzo 2005-mayo2006. (Disponible en: http://www.mortalidadmaterna.com.mx/docs/Monitoreo.pdf).

3. María del Carmen Elú, "Iniciativa para una Maternidad sin riesgos en México", en Graciela Freyermuth y María Manca, Luna golpeada. Morir durante la maternidad: investigaciones, acciones y atención médica en Chiapas y otras experiencias en torno a la mortalidad materna, Comité promotor por una maternidad sin riesgos en México, México, 2000, p. 14.

4. Langer Ana. "La Mortalidad materna en México: la contribución del aborto inducido", en Ortiz A. (ed.), Razones y pasiones en torno al aborto, México, Edamex/Population Council, 1994, pp.149-153.

5. Organización de las Naciones Unidas. Objetivos de Desarrollo del Milenio. (Disponible en: http://www.objetivosdelmilenio.org.mx/).

6. Bergonzoli G. Mortalidad materna y neonatal relación ODM-OPS. (Disponible en: www.lachealthsys.org/.../ _ODMs-OPS-Gustavo%20Bergonzoli.pps).

7. Lozano AR, Suárez VA. Mortalidad neonatal. Bol Med Hosp Infant Mex 2004;61:275-9)

8. American Academy of Pediatrics y American Heart Association. (2006). Reanimacion Neonatal. (5th ed).

9. Sistema Nacional de Información Estadística y Geográfica (INEGI) y Dirección de Informática y Geografía Médica. Núcleo de Acopio y Análisis de Información en Salud. INSP con datos obtenidos de la Secretaría de Salud. Se analizó a nivel estatal, con datos de la Encuesta Nacional de Salud 2000 y la Encuesta Nacional de Salud y Nutrición 2006.

10. Instituto Nacional de Estadística, Geografía e Informática (INEGI). Conteo 1995 y 2005, XII Censo General de Población y Vivienda 2000. Procesada por Instituto Nacional de Salud Pública INSP

11. Steven G. Gabbe, MD, Jennifer R. Niebyl, MD, Joe Leigh Simpson, MD, Henry Galan, MD, Laura Goetzl, MD, MPH, Eric R.M. Jauniaux, MD, PhD, and Mark Landon, MD Obstetrics: Normal and Problem Pregnancies, 5th Edition. Elsevier publishers. ISBN: 9780443069307

12. Deborah Maine Detours and shortcuts on the road to maternal mortality reduction Viewpoint. Lancet 2007; 370: 1380–82

13. Otchere SA, Kayo A. The challenges of improving emergency obstetric care in two rural districts in MaliInt J Gynaecol Obstet. 2007 Nov;99(2):173-82. Epub 2007 Sep 27

14. M. Kayongo, J. Butera, D. Mboninyibuka, B. Nyiransabimana, A. Ntezimana, V. Mukangamuje. Improving availability of EmOC services in Rwanda — CARE's experiences and lessons learned at Kabgayi Referral Hospital Int J Gynaecol Obstet. 2006 Mar;92(3):291-8.

15. Kayongo M, Esquiche E, Luna MR, Frias G, Vega-Centeno L, Bailey P Strengthening emergency obstetric care in Ayacucho, Peru Int J Gynaecol Obstet. 2006 Mar;92(3):299-307.

16. Department of Health. Working for patients. London: HMSO, 1989.

17. Kongnyuy EJ, Leigh B, van den Broek N Effect of audit and feedback on the availability, utilisation and quality of emergency obstetric care in three districts in Malawi Women Birth. 2008 Dec;21(4):149-55

18. Muffler N, Trabelssi Mel H, De Brouwere V. Scaling up clinical audits of obstetric cases in Morocco Trop Med Int Health. 2007 Oct;12(10):1248-57.

19. Cohen S.R., Cragin, L., Walker, D.M. datos aún no publicados

20. WHO/ICM/FIGO, 2005

21. Davis D, Thomson O'Brien MA, Freemantle, Wolf FM, PhD; Mazmanian P, Taylor-Vaisey. A Impact of Formal Continuing Medical Education Do Conferences, Workshops, Rounds, and Other Traditional Continuing Education Activities Change Physician Behavior or Health Care Outcomes? JAMA. 1999;282 (9):867-874.

22. Halamek LP,Kaegi DM, Gaba DM, Sowb YA, Smith BC, Smith BE, Howard SK Time for a New Paradigm in Pediatric Medical Education: Teaching Neonatal Resuscitation in a Simulated Delivery Room Environment PEDIATRICS Vol. 106 No. 4 October 2000, p. e45 ELECTRONIC ARTICLE

23. W R Hamman The complexity of team training: what we have learned from aviation and its applications to medicine Quality and Safety in Health Care 2004;13(Supplement 1):i72-i79; doi:10.1136/qshc.2004.009910 Copyright © 2004 by the BMJ Publishing Group Ltd.

24. SSM Health Care's Foundation of Safety and Care. STEPPS: Producing Effective Medical Teams to Achieve Optimal Patient Outcomes AHRQ Annual Conference Sept. 10, 2008

25. JamtvedtG, Young JM, KristoffersenDT, Thompson O'Brien MA, OxmanAD. Audit and feedback: effects on professional practice and health care outcomes. TheCochrane Database SystRev2003; 3: CD000259. DOI: 10.1002/14651858.CD000259.
